# Electrochemical Biosensors for Determination of Colorectal Tumor Biomarkers

**DOI:** 10.3390/mi11040411

**Published:** 2020-04-14

**Authors:** Jennifer Quinchia, Danilo Echeverri, Andrés Felipe Cruz-Pacheco, María Elena Maldonado, Jahir Orozco

**Affiliations:** 1 Max Planck Tandem Group in Nanobioengineering, University of Antioquia, Complejo Ruta N, Calle 67 No. 52-20, Medellín 050010, Colombia; jennifer.quinchia@udea.edu.co (J.Q.); danilo.echeverrih@udea.edu.co (D.E.); andres.cruz1@udea.edu.co (A.F.C.-P.); 2 Grupo Impacto de los Componentes Alimentarios en la Salud, School of Dietetics and Human Nutrition, University of Antioquia, A.A. 1226, Medellín 050010, Colombia; maria.maldonado@udea.edu.co

**Keywords:** colorectal cancer, electrochemical biosensors, biomarkers, *in vitro* diagnostic, point-of-care testing

## Abstract

The accurate determination of specific tumor markers associated with cancer with non-invasive or minimally invasive procedures is the most promising approach to improve the long-term survival of cancer patients and fight against the high incidence and mortality of this disease. Quantification of biomarkers at different stages of the disease can lead to an appropriate and instantaneous therapeutic action. In this context, the determination of biomarkers by electrochemical biosensors is at the forefront of cancer diagnosis research because of their unique features such as their versatility, fast response, accurate quantification, and amenability for multiplexing and miniaturization. In this review, after briefly discussing the relevant aspects and current challenges in the determination of colorectal tumor markers, it will critically summarize the development of electrochemical biosensors to date to this aim, highlighting the enormous potential of these devices to be incorporated into the clinical practice. Finally, it will focus on the remaining challenges and opportunities to bring electrochemical biosensors to the point-of-care testing.

## 1. Introduction

More than 1.8 million new cases of colorectal cancer (CRC) were diagnosed worldwide in 2018, positioning as the third type of cancer of highest incidence in both men and women [1]. With 880,792 deaths reported to 2018, CRC was the second cause of cancer-related death [1]. 

In the last years, the screening/diagnostic strategies are evolving toward minimally invasive and easy-to-use tests, intending to increase patient uptake and decrease the mortality rate. In this sense, the *in vitro* diagnostic (IVD) of tumor markers is the focal point of research in cancer detection. From the first genetic model of colorectal tumorigenesis proposed by Fearon and Vogelstein in 1990 [2] until today, when it is known that the transformation of adenoma to carcinoma is driven not only by genetic alteration but epigenetic alterations [3], many tumor markers have been proposed to describe this complex process [4,5,6,7,8,9,10,11]. The most recent findings regarding molecular events along the adenoma–carcinoma sequence urgently demand the development of detection methodologies and strategies that allow the simultaneous determination of tumor markers of different molecular nature with simple protocols and suitable for point-of-care (POC) testing. 

To pave the way to solve this need, the detection and quantification of biomarkers by electrochemical biosensors are at the forefront of tumor cancer determination research because of their unique features such as versatility, fast response, accurate quantification, and amenability for multiplexing and miniaturization. Such remarkable features make electrochemical biosensors hold promise for the development of POC testing devices for cancer monitoring.

In the first section of this review, we will explain why the transition toward the diagnosis of CRC based on tumor biomarkers is currently necessary considering its potential for clinical diagnosis, prognosis, and follow-up of treatment, discussing the requirements for biomarkers determination, the currently available methodologies, and their limitations. Hereafter, through an exhaustive and critical summary of the electrochemical biosensors developed to date, we will show how their principles of detection and quantification make them a promising alternative for the *in vitro* diagnostics and monitoring of tumor biomarkers. Finally, we will point out the remaining questions and bottlenecks that further works need to solve in this field and the impact that these technologies may have on the routine clinical analysis.

## 2. Challenges in CRC Diagnosis, Prognosis, and Therapeutic Response Evaluation

CRC is a malignancy with high incidence and mortality rates worldwide [1]. Although an increase in both incidence and mortality is projected [12], the decrease in CRC-related deaths is linked with the early detection of the disease and, therefore, adequate clinical management [13]. Making an accurate diagnosis and assertive treatment in the early stages, the five-year survival rate of patients can reach values in the order of 90%, while in the late stages, it decreases significantly (about 14% for the metastatic stage) [5,14], which confirm that early detection saves lives.

The window in the adenoma–carcinoma sequence where the early detection influences CRC survival includes from the cancer-initiating event to the formation of localized CRC [14]. In these stages, patients are usually asymptomatic, and screening methods are the best way to get outcomes of the disease. The two CRC screening modalities involve stool-based tests and visual (structural) exams. Stool-based strategies (i.e., FIT: Fecal Immunochemical Test and gFOBT: Guaiac-based Fecal Occult Blood Test) identify hidden blood in the stool. These methods are considered non-invasive, easy to use, affordable, and flexible for screening in general populations [15,16,17,18], but they usually show false-positive results because hidden blood in the stool can be related with several triggering pathologies.

On the other hand, direct structural exams look for atypical areas in the structure of the colon and rectum. Colonoscopy, Flexible Sigmoidoscopy (FSIG), and Computed Tomographic Colonography (CTC) are part of this group. Colonoscopy is the gold standard of CRC screening tests. It examines the colon and rectum in a single session for the identification and elimination of colorectal polyps in non-metastatic cancers. FSIG looks at only about one-third of the colon and removes the polyps of these sections. CTC examines the structure of the rectum or colon in a non-invasive manner without the possibility of eliminating polyps [15,16,17,18]. These tests are also used to diagnose symptomatic people or follow-up to patients when screening tests show abnormal outcomes [19].

Despite the variety of screening and diagnostic approaches, more than 50% of CRC-related deaths occur in the unscreened population. The effectiveness of these tools is jeopardized by a multitude of factors, including the limitations of test performance (sensibility and specificity) in some screening/diagnosis tests, their invasive nature, unfriendly bowel preparation, and centralized diagnosis, among others. The weaknesses of these approaches and the urgent need for taking immediate clinical management decisions to improve patient treatment has led to the reconsideration of the CRC screening/diagnosis strategies. Doctors are moving from conventional tests such as colonoscopy to easy-to-use screening/diagnostic tests, but they are trying to keep the selectivity and specificity achieved with the gold standard. Looking for this transition, the development of non-invasive screening/diagnostic tests based on the determination of tumor markers is an object of study in cancer diagnosis research.

The National Cancer Institute (NCI) defines a tumor marker (biomarker) as “anything present in or produced by cancer cells or other cells of the body in response to cancer or certain benign (noncancerous) conditions that provides information about cancer, such as how aggressive it is, whether it can be treated with targeted therapy, or whether it is responding to treatment” [20]. Hence, biomarkers can be classified according to their clinical use in three main groups: (i) diagnostic biomarkers for risk stratification and early detection, (ii) prognosis biomarkers for giving a signal of the likely disease progression, and (iii) therapy response evaluation biomarkers for monitoring how the patient responds to a specific treatment [15,21]. 

It is known that cancer is a multifaceted and multi-stage disease whose progression from its initial stage to its metastatic stage comprises a complex variety of genetic or epigenetic alterations [3], generating abnormalities in transcription, translation, and protein expression [22]. Tumor biomarkers include nucleic acids (i.e., messenger RNA, non-coding RNA, DNA), peptides, proteins (e.g., enzymes, hormones, antibodies, conjugated proteins, receptors), among other categories, which may be found in tumor tissues, circulation (blood, serum, or plasma), or excretions/secretions (stools, urine, sputum, saliva) [23]. Advances in the identification of CRC-correlated biomarkers have allowed their implementation in the clinical routine to determine them according to their clinical purpose. Table 1 summarizes the type and the CRC biomarkers according to their clinical use and shows them at different molecular levels and matrices, as well as their commercial availability. 

The current trend in clinical practice is directed to the determination of biomarkers in blood because this biological matrix contains circulating biomarkers such as proteins, exosomes, nucleosomes, circulating cancer cells (CTCs), and circulating cell-free nucleic acids, which provides complete information on the progression of the neoplasm [6,14,24]. Besides, blood-based tests offer a minimally invasive sampling and the possibility of a patient-friendly approach, improving patient uptake and impacting on the CRC survival rate [14]. Many reports have registered blood biomarkers of different molecular natures and classified them depending on their role in CRC monitoring [4,5,6,7,8,9,10,11]. Table 2 compiles this information in a list of blood-based biomarkers of CRC, classifying them according to their type and clinical purpose. As shown in this table, a high number of biomarker candidates have been proposed for CRC monitoring due to their altered expression (down- or up-regulation, hypo- or hypermethylation) in patients concerning healthy individuals. Yet, the reliability of these biomolecules needs to be vastly probed before adopting them as biomarkers in a clinical laboratory. 

Although advances in the classification of CRC biomarkers according to their clinical purpose have allowed progress toward an *in vitro* and stratified disease diagnostic, current techniques for detecting tumor markers limit their evolution toward the POC testing. Among the technologies applied in clinical practice for the detection and quantification of biomarkers, the Enzyme-Linked Immunosorbent Assay (ELISA) for detecting proteins and quantitative Polymerase Chain Reaction (qPCR) for nucleic acid stand out. These strategies are well suited for the sensitive detection of biomarkers of a single molecular level in certain types of samples or pretreated samples, but they have limited ability for detecting multiple-level biomarkers. Other disadvantages involve multi-steps and time-consuming processes, the demand for specialized operating staff, high cost, and incompatibility with miniaturization. 

To overcome the barriers of conventional methods for the clinical biomarkers determination and to achieve new approaches for IVD, it is necessary (i) to detect and quantitate biomarkers at different stages of the disease in a cost-effective manner to take adequate clinical management and improve patient treatment, (ii) to develop new diagnostic tools that are able to establish a reliable diagnosis at the time and place of patient care (POC testing), and (iii) to develop diagnostic strategies with multiplexed capacity (determination of biomarkers at different clinical ranges and molecular levels) in a single test. In this context, features of electrochemical biosensors make them promising alternatives with respect to conventional methods in terms of portability and miniaturization capabilities, real-time response, and capacity for the specific and simultaneous detection of tumor biomarkers of different natures.

## 3. Nanobioengineered Electrochemical Biosensors

According to the International Union of Pure and Applied Chemistry (IUPAC), a biosensor is a self-contained integral device that can provide specific quantitative/semi-quantitative analytical information using a biological recognition element (biochemical receptor) in direct spatial contact with a transducer element [49]. Biosensors have been developed with a variety of biochemical receptors, including enzymes, proteins, antibodies, nucleic acids, cells, tissues, or receptor molecules [50,51,52,53]. The bioreceptor interacts selectively with a target analyte, generating a biochemical response that the transducer converts into a measurable signal, which is analyte concentration-dependent [49]. Then, the signal is collected by a signal processor and amplified before being displayed in an electronic display system. 

Biosensors can be categorized based on the physicochemical principle used by the transducer to transform the information collected from the bioreceptor–analyte recognition event. Thus, the transducer can be electrochemical, optical, piezoelectric, thermoelectric, etc. Electrochemical biosensors are especially attractive because the inherent combination of the robustness from the transducer platform with the selectivity from the biological component may lead to highly sensitive devices with a wide dynamic linear range and low limit of detection (LOD). Furthermore, apart from the manufacture of electrochemical biosensors being affordable and straightforward, their short response times and ease miniaturization allow for the development of portable devices that need a few sample volumes for their operation [54,55]. 

Electrochemical biosensors can be also classified according to the electrochemical technique that they use to register the response. The biosensing field commonly utilizes several electroanalytical techniques. For example, voltammetric techniques include cyclic voltammetry (CV), differential pulse voltammetry (DPV), and square wave voltammetry (SWV), as well as amperometry, potentiometry, and electrochemical impedance spectroscopy (EIS) [56]. In voltammetric methods, a time-dependent potential is applied in an electrochemical cell, and the resulting current is measured as a function of the applied potential [57]. Amperometric and voltammetric biosensors are based on the application of a potential between a working electrode (WE) and a reference electrode (RE) that generates a current as a result of the oxidation/reduction processes of the electroactive species present in a solution at the electrode surface. A counter electrode closes the circuit and facilitates the electron flow through. Voltammetric biosensors measure the current when the potential is swiped at a constant speed in a potential window, where the resulting peak is often proportional to the analyte concentration [58]. The amperometric configuration allows for measuring current changes at a set potential, being selective, since the oxidation/reduction potential is characteristic of the analyzed species. DPV and SWV are used in biosensing to lower the capacitive current and increase the sensitivity [59]. A potentiometric biosensor is a device incorporating a biological sensing element connected to an electrochemical transducer whose analytical signal is an electrical potential [60]. The potential of the electrochemical cell is measured under static conditions with negligible current flow [57].

EIS measures the resistive and capacitive properties of an interface after disturbing the system by a sinusoidal AC excitation signal of a small amplitude (approximately 2–10 mV). The frequency is changed over a wide range to obtain an impedance spectrum. Then, the in-phase and out-of-phase current responses are measured to determine the resistive and capacitive components of the circuit. At high frequency, the migration rate of the redox species to the electrode surface becomes rate limiting, and thus analytes that block access to the electrode surface can generate a frequency-dependent phase lag between the AC voltage and the current [58,61]. The voltage–current response in the range of frequencies analyzed can be studied by fitting the experimental data with a specific equivalent circuit that explains the electrochemical behavior of the electrode surface [62]. The equivalent circuit consists of different elements that represent the electrochemical system, e.g., the bulk solution resistance (R_s_), the double-layer capacitance (C_dl_), the Warburg’s impedance (Z_w_), and the interfacial electron transfer resistance (R_ct_). EIS is a label-free technique commonly applied to detect analytes down to a single-molecule (attomolar) level [63,64,65]. In this context, EIS-based biosensors could be more sensitive electrochemical devices as compared to voltammetric and amperometric biosensors [66].

**Figure 1:**
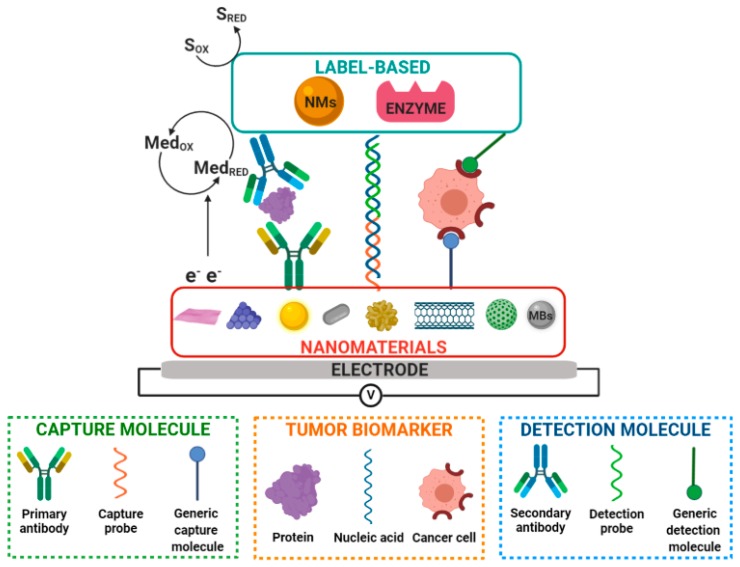
Schematics illustration of nanomaterial-based electrochemical biosensors. S_RED_ and S_OX_ and Med_RED_ and Med_OX_ indicate substrate reduction and oxidation and mediator reduction and oxidation, respectively. NMs and MBs indicate nanomaterials and magnetic beads, respectively.

Electrochemical biosensors can be also distinguished as catalytic or affinity-based biosensors. Catalytic-type biosensors employ enzymes, cells, or microbes to generate electroactive species that are reduced or oxidized on the electrode surface and are correlated to the amount of analyte. The most used enzymes in the manufacture of these biosensors are glucose oxidase, alkaline phosphatase (ALP), horseradish peroxidase (HRP), and catalase. [67]. Electrochemical biosensors of biochemical affinity study specific biological recognition interactions such as antigen–antibody binding and nucleic acid hybridization reactions, primarily triggering a measurable electrochemical response (Figure 1). These electrochemical biosensors are usually the most used in the detection of a large number of biomolecules of clinical relevance due to their high specificity [68].

Affinity biosensors can be also classified as label-free and label-based biosensors [69]. The electrochemical signal collected at the electrode when the bioreceptor–analyte biological recognition event occurs can be measured using strategies based on tags. In this case, the biosensors are called label-based biosensors. The tags can vary from enzymes with a detectable product to organic molecules or nanomaterials with electrocatalytic properties. In some cases, signal labels are necessary for the system to achieve readable output signals [70]. For this goal, various labeling strategies are used to amplify the detection signal in biosensors technology. Labeling may involve avidin–biotin conjugation along with redox enzymes, as well as covalent attachment, intercalation, or the electrostatic interaction of small molecules/particles/ions with the biorecognition elements, which are responsible for generating the electrochemical signal [71].

In contrast to conventional label-based biosensors, label-free biosensors are based solely on the measurement of changes of the electrical signal at the electrode surface when the interaction of the target analyte and the biological recognition element occurs [72]. Label-free biosensors directly transduce a molecular binding event into a physically measurable quantity, i.e., without the need for an additional antibody, enzymatic, or electroactive label, or any other amplification strategy, to provide a response that is proportional to the concentration of bound molecules [71]. Transduction is commonly achieved by measuring a physical property change, such for example, the charge transfer resistance (R_ct_) by EIS [73].

Additional benefits of the electrochemical biosensors include simplicity in the required equipment, cost-efficiency, and easiness of adaptation of electrode characteristics and surface chemistry for specific applications [74]. 

With the progress in the fields of nanoscience and nanotechnology, a wide range of nanomaterials with excellent chemical, physical, and mechanical properties have been developed and adapted in biosensing platforms. They include several types of nanomaterials such as noble metals [75], metal oxides [76], metal chalcogenides [77], magnetic nanoparticles (NPs) [78], carbon-based nanomaterials [79], conductive polymers [80], etc. Among them, metal nanostructures have been attractive in the design of highly sensitive electrochemical biosensors. The noble metal nanostructures including Au, Ag, Pt, Pd, Ni, etc. have been found to play a significant role in the development of biosensors to fulfill the increasing demand of highly specific and highly sensitive diagnostics devices. The extraordinary properties of nanostructured noble metals lead to a great improvement of the electrochemical biosensors due to the high surface energy, reduced size, and fast electron transfer possess, which could facilitate the electrochemical reactions at the electrode–solution interface. Recently, the nanostructured metal oxides have also aroused as much interest as tagging elements and surface modifiers, thus increasing the electroactive surface area and favoring the electron transfer at the transducer surface [81]. Metal oxides have a high surface area-to-volume ratio, low toxicity, chemical stability, and biocompatibility, as well as being environmentally friendly. Metal oxides also show the fast electron transfer properties required to improve nanomaterial-based biosensors’ performance. Recently, some metal oxides have also been used as labels that mimic the enzymatic activity due to the catalytic similarity that they present with peroxidases [82]. In this context, the inorganic nanostructures such as iron oxide (Fe_2_O_3_), zinc oxide (ZnO), nickel oxide (NiO), zirconium oxide (ZrO_2_), cerium oxide (CeO_2_), titanium dioxide (TiO_2_), silicon dioxide (SiO_2_), etc. have opened new opportunities thanks to their multifunctional properties [83]. These nanostructured metal oxides can be utilized for the fabrication of electrochemical biosensors to quantify biomarkers with excellent performance including high sensitivity, cost-effectiveness, and a low LOD. The electron transfer properties of metal oxides are very significant to understand the carrier transport mechanism for electrochemical transducers fabrication. 

The use of magnetic nanostructures constitutes an innovative approach to the development of biosensor platforms. Commonly, superparamagnetic nanoparticles of iron oxide in the form of magnetite (Fe_3_O_4_) or maghemite (γ-Fe_2_O_3_) are widely used. The basic strategy in analytical applications focuses on the magnetic preconcentration of an analyte at an electrode surface by applying an external magnetic field. In addition to the inherent magnetic force, superparamagnetic nanoparticles can also improve the analytical signal due to the oxidation/reduction processes that may decrease the signal/noise ratio [84]. Nanocomposite materials have also been extensively explored, since it is possible to exploit the properties of the different individual components that they are made up with the improved features of the resultant nanocomposite, which are of great utility in the detection of a variety of biomarkers [85,86]. In nanocomposites, at least one of its components is at the nanoscale. According to the matrix material, the nanocomposites are classified into polymeric, metal, and ceramic-matrix compounds. Polymeric matrix nanocomposite materials, especially semiconductor polymers, have been widely used in biosensors as an intermediate layer between biological molecules and electrodes used for signal reading [87]. Such corrosion-free matrixes of simple synthesis enjoy efficient electron transfer capacity, high biocompatibility, and environmental stability.

The variety of nanomaterials along with the different methodologies for their synthesis allow generating a myriad of nanostructure shapes such as nanotubes, nanospheres, nanosheets, nanorods, nanowires, nanowhiskers, nanoflakes, nanocubes, and nanopillars, among others [88]. Likewise, the dimensional similarity of the nanostructures with the biological molecules provides the opportunity to immobilize more biomolecules without losing their function. Besides, stability, biocompatibility, and the advantage of modulating the surface of nanomaterials make them easy to conjugate multiple chemical species, biological species, and polymeric materials [89]. Therefore, control of the size, structure, chemical composition, shape, and modification of the surface of the nanostructures can affect the electrical and physicochemical performance of both the transducer platforms and the signal tags. Thus, the development of methodologies for the fabrication of nanoscale entities by top–down and bottom–up methods controlling intrinsic and extrinsic characteristics will provide greater efficiency in the detection of tumor biomarkers with electrochemical devices.

The appeal of such carefully designed nanostructures, when incorporated in biosensing platforms, is related to their intrinsic unique features that include a high surface–volume ratio, the possibility to act as effective immobilization matrices for the immobilization of a high number of biomolecules, and their resultant improved electron transfer and electrocatalytic activity ability [90]. Functional nanomaterials have the potential to produce a synergistic effect among catalytic activity, conductivity, and biocompatibility, which results in improved signal transduction and amplification of the biorecognition events, and it also contributes to the development of highly sensitive and specific devices [91]. 

## 4. Electrochemical Biosensing of Biomarkers Associated with CRC

During the process in which an adenoma is transformed in a carcinoma, genetic-type (e.g., mutations, deletions, and insertions, among others in the chromosomes) [21,92] or epigenetic-type alterations (gene silencing or activation by the hypo/hypermethylation mechanism, change in the expression of microRNA, and histones modification, among others) [93,94] have a variety of effects on the downstream transcription and transduction products that modify both the cells and tumoral microenvironment. Based on the heterogeneity of molecular events associated with CRC, the electrochemical biosensors described so far in the literature determine nucleic acids, proteins, and tumor cells through biomarker–recognition molecule affinity interactions. Electrochemical bioassays involve specific single oligonucleotide strands of DNA (both linear and hairpin probes), aptamers, antibodies, lectins, or small organic molecules as recognition biomolecules in either label-based or label-free bioassay formats. Some publications are focused on studying capture biomolecules to promote highly specific and selective recognition events. Others address the development of new amplification strategies for the detection of colorectal tumor biomarkers in the different clinical ranges by using enzymes or nanomaterial-modified electrodes, advanced labels, and carriers of proteins/electroactive materials, among others. They are also intended for the implementation of microfabrication processes, incorporation of microfluidic systems, and use of flexible substrates and screen-printed electrodes (SPEs) coupled to electrochemical biosensing systems. The ultimate goal is to achieve devices with outstanding characteristics in terms of high efficiency, the minimization of nonspecific interactions, and high sensitivity, as well as versatility, portability, and the minimization of reagents and samples. The following subsections discuss the electrochemical biosensing strategies developed so far for CRC diagnosis according to the type of biomarker (nucleic acids, proteins, and tumor cells) detected.

### 4.1. Electrochemical Biosensing of Nucleic Acid Biomarkers of CRC

Most studies that report the electrochemical determination of nucleic acids as CRC biomarkers include specific genes, mutant DNA, methylated/hidroxilated DNA, and specific microRNA (Table 3). The first electrochemical detection of a CRC-related gene was by DPV [95]. A capture DNA was immobilized on a CeO_2_/Chitosan (CHIT) composite film deposited on top of a glassy carbon electrode (GCE) by electrostatic interactions. After a hybridization reaction with the complementary target, a reduction signal of methylene blue was recorded to determine the amount of the DNA biomarker with a linear range between 1.59 × 10^−11^ and 1.16 × 10^−7^ mol L^−1^.

KRAS (Kirsten Rat Sarcoma viral oncogene homolog) is a proto-oncogene that contains information for the synthesis of the GTPase KRAS protein. This protein is a central mediator in cell-signaling pathways related to cell growth and plays a critical role in cell maturation, proliferation, cell death, and differentiation [96,97]. Although the expression of the wild-type KRAS gene does not ensure the effectivity of the therapy based on epidermal growth factor receptor (EGFR) inhibition, this biomarker is currently measured in a companion device approved by the Food and Drug Administration (FDA) [38]. Zhijie Wang et al. [98] developed the first electrochemical biosensor for the detection of the KRAS gene, which is highly associated with CRC. A sandwich-type format involved the immobilization of a binary self-assembled monolayer (SAM) composed of a thiolated specific capture DNA probe and thioglycolic acid as a spacer on a gold electrode. The sandwich was achieved after the hybridization reaction with the target and an HRP-labeled DNA signal probe. The genosensor produced an amperometric response in a concentration-dependent manner, with a linear range from 1.17 × 10^−11^ to 1.17 × 10^−7^ M and a LOD of 5.85 × 10^−12^ M with the hydroquinone/hydrogen peroxide (HQ/H_2_O_2_) system.

Later, Xiaoying Wang et al. [99] developed a sandwich-type genosensor based on a multiple signal amplification strategy. Nanofibers of carboxylated multi-walled carbon nanotubes (MWCNTs) doped with nylon 6 (PA6) served as the nanosized backbone for the electropolymerization of thionine (PTH) over GCE. The functional platform (MWCNTs-PA6-PTH) was used for the electrostatic immobilization of a single-stranded DNA1 capture probe (ssDNA1). The KRAS gene hybridized simultaneously with ssDNA1 and a gold nanoparticle (AuNP)-labeled ssDNA2 signal probe (AuNPs-ssDNA2). The formation of network-like thiocyanuric acid/AuNP (TA/AuNPs) served as the amplification strategy. The reduction of AuNPs in an acidic medium (AuCl_4_^−^) was quantified by DPV, whose signal response was KRAS gene concentration-dependent from 0.1 to 100 pM, with a LOD of 30 fM. The developed biosensor was tested in SW480 CRC cell lysates, which had results that were consistent with those from the analysis of the High-Resolution Melt (HRM) curve after amplification by PCR. 

Ahmed Jassim Muklive Al-Ogaidia and co-workers [100] reported voltammetric biosensors based on carbon matrices modified with either phthalocyanine-boron dipyrromethene (BODIPY) dye or azulene (A1: 2,6-bis((E)-2-(furan-2-yl)vinyl)-4-(4,6,8-trimethylazulen-1-yl)pyridine and A2: 2,6-bis((E)-2-(thiophen-3-yl)vinyl)-4-(4,6,8-trimethylazulen-1-yl)pyridine) composites as working electrode-based carbon matrices. The biosensors based on phthalocyanine-BODIPY, A1/PtTiO_2_-reduced graphene oxide, and A2/PtTiO_2_-reduced graphene oxide detected 2.06 × 10^−6^, 8.67 × 10^−10^, and 2.94 × 10^−5^ µg mL^−1^ of the KRAS gene in a linear concentration range from 1.54 × 10^−4^ to 1.92 × 10^−2^, from 3.07 × 10^−7^ to 3.84 × 10^−3^, and from 3.84 × 10^−8^ to 0.48 µg mL^−1^, respectively. The recovery of KRAS in spiked and in whole-blood samples suggests that the proposed biosensors have considerable potential as new devices to assess the KRAS gene levels. 

**Figure 2:**
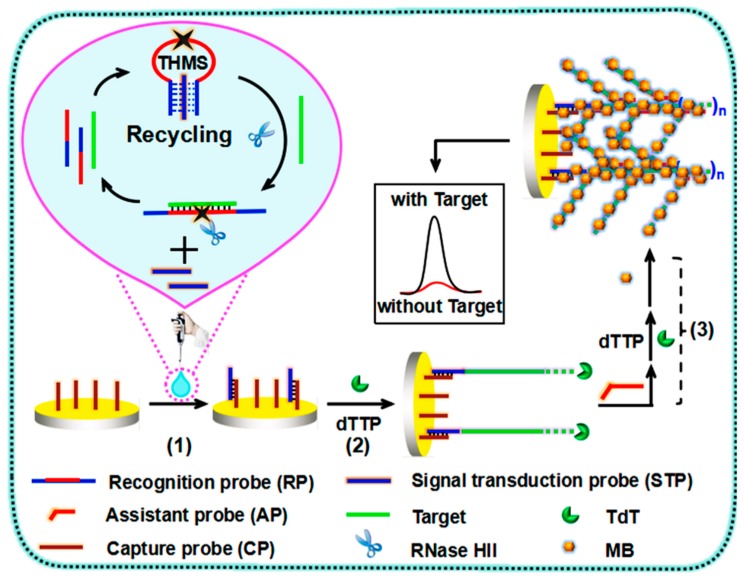
Schematic illustration of the dual enzyme-assisted multiple amplification for KRAS G12DM electrochemical determination. Reprinted from [106] with permission. Copyright © 2018, Elsevier B.V.

Aimed to predict better the therapeutic response against EGFR, some researchers analyzed point mutations in the KRAS gene, because its mutated versions are associated with the inefficiency of the cetuximab/panitumumab-based therapy [36,37,38,39,101,102]. KRAS mutations have been noted in almost 40% of all CRC, from which about 95% was found in codons 12 and 13 [101,103]. KRAS G12D (sometimes wrongly called KRAS G12DM) is the most reported KRAS point mutation in CRC [104,105]. This mutation implies the transition from guanine to adenine (G → A) in codon 12 with the substitution of aspartic acid by glycine on the downstream protein. Testing this mutation, among others, codon 12 and 13 in the KRAS gene, is now put in practice [39,40]. Hua-Feng Wang et al. [106] developed an ultrasensitive label-free biosensor based in a dual enzyme-assisted multiple amplification (RNase HII: Ribonuclease HII, and TdT: terminal deoxynucleotidyl transferase). The principle of the biosensor is schematically illustrated in Figure 2 and consists of three key steps. (1) A specially designed triple-helix molecular switch (THMS) employed as both a molecular recognition and signal transduction element to realize the RNase HII-assisted homogenous target recycling amplification and release numerous signal transduction probes (STP). (2) The released STP hybridized a thiolated capture probe attached to a gold electrode and triggered the TdT-mediated DNA polymerase to form a long single-stranded DNA between the STP and the target. (3) The second step of the TdT-mediated extension between DNA targets through a designed assistant probe (AP) generated a long stable DNA dendritic structure, which was decorated with the redox-active methylene blue. This approach detected above 2.4 aM of the target, with a linear range from 0.01 fM to 1 pM. The comparison of DPV responses of the biosensors with the single and dual enzyme-assisted amplification strategies demonstrated that it extends the linear range and decreases the LOD by one order of magnitude, respectively. The developed biosensor discriminated between the concentration of KRAS G12DM of plasma samples collected from five CRC patients and five plasma samples from healthy individuals. 

**Figure 3:**
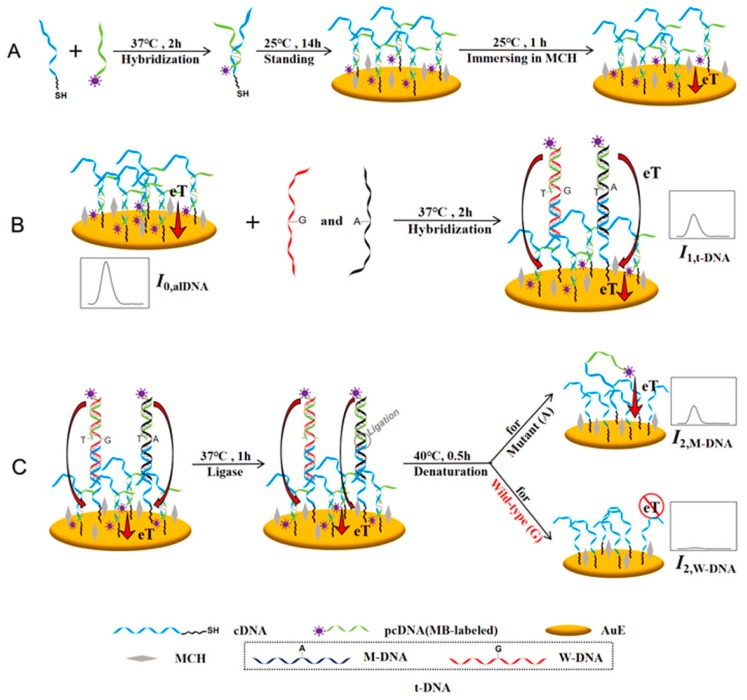
Scheme of the biosensor design to KRAS G12D point mutation level determination. (**A**) The formation of the anchor-like DNA (alDNA) sensing surface, (**B**) the detection mechanism of the total DNA (t-DNA) and (**C**) mutant DNA (M-DNA). Reprinted from [107] with permission. Copyright © 2019, Elsevier B.V.

Determination of both mutant (KRAS G12D) and wild-type KRAS genes on one chip was possible by a novel anchor-like DNA (alDNA), in which the KRAS G12D point mutation level (the concentration ratio of the specific KRAS point mutant DNA (M-DNA) to the total DNA (t-DNA; mutant wild-type DNAs)) was established [107]. In this bioassay (Figure 3), the alDNA was formatted by a hybridization reaction between the thiolated DNA (cDNA) immobilized on a gold electrode and methylene blue-labeled DNA (pcDNA). The unmatched nucleotides in cDNA and the whole sequence in pcDNA captured the t-DNA in a sandwich-type format, and the resulting current by SWV was employed for the quantitative detection of t-DNA. Afterward, the cDNA and pcDNA were linked by a DNA ligase in the M-DNA due to the total bases match, followed by a denaturalization process. As a result, the current from pcDNA was only due to M-DNA. The dynamic linear ranges were established from 0.1 pM to 10 nM for t-DNA and from 100 pM to 10 nM for M-DNA, respectively. The recovery experiments of t-DNA-spiked human serum samples revealed the high accuracy of the developed method. 

The BRAF gene (referred to as proto-oncogene B-Raf or v-Raf murine sarcoma viral oncogene homolog B) is another essential proto-oncogene associated with the cell grown pathway, which is also involved in cell proliferation, differentiation, and transcriptional regulation [108]. BRAF V600E is the most critical BRAF gene mutation in CRC [105,109]. This point mutation is associated with the substitution of thymine with adenine (T → A) in codon 600, and the last change of valine by glutamate acid in the transduction product [110]. The role of BRAF V600E has been evaluated in the therapeutic response of binimetinib, cetuximab, encorafenib, panitumumab, dabrafenib, trametinib, and vemurafenib [108,109]. Since both BRAF and KRAS are involved in MAP kinase signaling [96,108], some authors have tried to find some relation between these biomarkers and their mutations [111]. The electrochemical determination of BRAF V600E was by DPV after the amplification-refractory mutation system (ARMS) [110]. First, the ARMS reaction was developed with a thiolated forward primer. Then, the products were tagged with biotin molecules by the incorporation of biotinylated dCTP in the reaction. The thiolated amplicon was immobilized on Fe_3_O_4_/Au NP by the formation of the gold–sulfur bond. Then, ALPs were loaded on the amplicon through biotin–streptavidin–ALPs interactions, resulting in a multienzyme-labeled bioconjugate. The sandwich magneto-bioconjugate was magnetically attracted to the surface of screen-printed carbon electrodes (SPCE), and the oxidation current of ascorbic acid (AA) measured by DVP was proportional to the V600E-mutant alleles extent in the 50–0.8% range. This approach resulted in being more sensitive in the determination of BRAF V600E in CRC cell-line HT29 than DNA sequencing and agarose gel electrophoresis. 

The invasion of cancer cells to distant organs or tissues is an important event that occurs in the evolutionary process of cancer. Cell adhesion biomolecules mediate these processes. Therefore, changes in the expression of the coding gene of cell adhesion molecules have implications in tumor progression, from the detachment of tumor cells from the primary site until the formation of the secondary lesions [112]. Carcinoembryonic antigen-related cell adhesion molecule 5 (CEACAM5) is overexpressed in CRC [113,114]. The pre-warning and prognostic nature of this protein motivate the determination of its coding gene. An electrochemical sensor was developed for the detection of CEACAM5 based on thin-film technology using silicon dioxide as a substrate [114]. The three-electrode system consisted of (i) a platinum semicircular counter electrode with 200-nm thickness deposited onto the silicon substrate by direct current (DC) sputtering, (ii) a gold disk with 100-nm thickness and a 350-µm diameter deposited by radio frequency (RF) sputtering as a working electrode, and (iii) an external Ag/AgCl reference electrode. The gold electrode was biofunctionalized with mixed SAMs of a thiolated capture probe and 6-mercapto-1-hexanol. The biosensor was characterized by EIS and CV. The specificity of the designed capture probe was demonstrated by qPCR, analyzing the CEACAM5 expression (obtained by retrotranscription of RNA) in a non-metastatic CRC cell line (WIDR), in metastatic CRC cell-lines (T84 and LOVO), in a liver cancer cell-line (HEPG2), and in peripheral blood lymphocytes from healthy individuals. The study did not report quantitative results.

**Figure 4:**
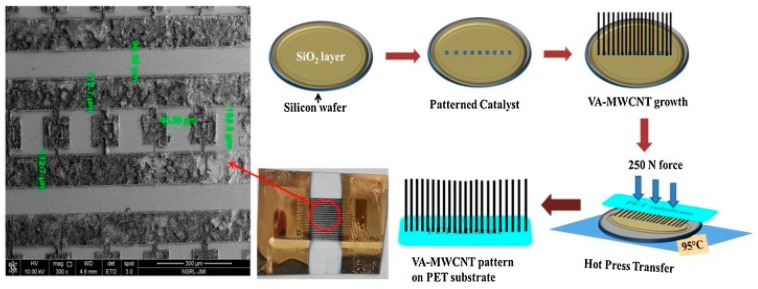
Representation of vertically aligned multi-walled carbon nanotube (VA-MWCNT) pattern transferred onto a polyethylene terephthalate substrate by the hot press technology. Reprinted from [113] with permission. Copyright © 2019, Elsevier B.V.

In a more recent study, Payal Gulati et al. [113] used polyethylene terephthalate (PET) as a substrate to design a flexible electrochemical device. A pattern of vertically aligned MWCNT (VA-MWCNT) synthesized onto Si/SiO_2_ solid substrate by thermal chemical vapor deposition (CVD) was further transferred onto the flexible material through the hot press technique (Figure 4). The NH_2_-labeled capture probe was covalently immobilized on the VA-MWCNT after the substrate oxidation by oxygen plasma treatment. The designed biosensor provided a linear range within 50 to 250 µM with a LOD of 0.92 µM for the synthetic target, which was calculated form CV with methylene blue as a redox mediator. The usefulness of the genosensor was demonstrated by detecting the CEACAM5 obtained from the RNA retrotranscript extracted from the T84 CRC cell-line.

Unlike genetic-type alterations, epigenetic-type alterations do not change the DNA sequence. However, the inactivation of tumor suppressor genes and the activation of oncogenes occur by other mechanisms, including DNA methylation (hypo- or hypermethylation), the modification of histones, and the dysregulation of noncoding RNA [93,94]. The methylation of DNA is one of the most important epigenetic mechanisms, in which a region rich in the guanine-cytosine (G-C) sequence (called the CpG island) can be hypo- or hyper-methylated, impacting on the transcriptional process [4]. DNA methyltransferases catalyze the covalent binding of the methyl group at the fifth carbon of the cytosine ring 5-methylcytosine (5-mC). After, 5-mC can be oxidized to 5-hydroxymethylcytosine (5-hmC) by ten-eleven translocation (TET) methylcytosine dioxygenase oxidase [115]. The O^6^-methylguanine-DNA methyltransferase (MGMT) gene, whose functional product removes alkylating groups from O^6^-guanine, is an important example of this epigenetic mechanism [94,116]. This gene is frequently hypermethylated in CRC [4].

Povedano and co-authors [115] developed the first electrochemical bioplatform to detect methylation events at localized sites with single-base sensitivity. The strategy is based on streptavidin–modified magnetic beads with a biotinylated-DNA capture probe, followed by hybridization with a synthetic target DNA sequence with a single 5-hydroxymethylcytosine (5-hmC) in the promoter region of the MGMT gene, the recognition of the methylation event by a specific antibody (anti-5-hmC), and further conjugation with one bioreagent for the signal amplification. Bioreagents were either bacterial antibody binding protein (ProtA) or Histostar, which were conjugated with HRP molecules. The electrochemical detection was achieved by amperometry by using the H_2_O_2_/HQ system at disposable SCPEs in a linear range of 77–7500 pM (LOD 23 pM) and 44–5000 pM (LOD 13 pM) for amplification with each bioreagent, respectively. The biosensor detected 5-hmC methylation in 10 ng of gDNA extracted from tumor cells (SW480 and SW620) and paraffin-embedded tissues of CRC patients and the presence of this methylation type in the MGMT gene promoter region by using an amount of sample 10-fold lower than that of other reported electrochemical platforms. Its versatility, rapid execution, and ease of implementation at low cost were remarkable.

**Figure 5:**
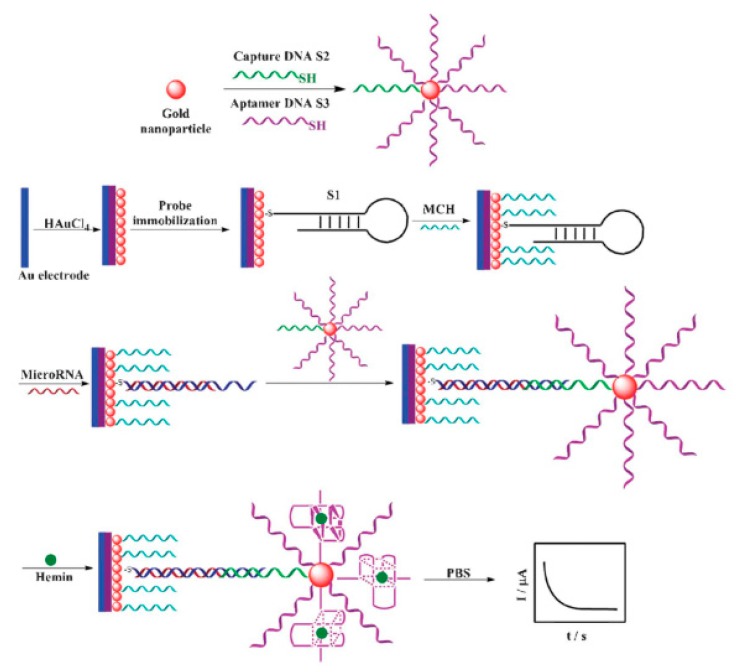
Schematic representation of miRNA-21 detection by a label-free electrochemical sensing approach making use of a hemin-G–quadruplex complex as the amplification element. Reprinted from [118] with permission. Copyright © 2012, Royal Society of Chemistry.

MicroRNAs (miRNAs) are other relevant epigenetic biomarkers that are considered as reliable tumor biomarkers that link to cancer and its progress [117]. These nucleic acids are small non-coding RNA molecules (19–25 ribonucleotides) that mediate post-transcriptional gene expression through either messenger RNA (mRNA) degradation or stopping their transduction into functional products [4,116,117]. In the same way as genes, microRNAs can act as oncogenes (oncomiRNA) or tumor-suppressive genes (tsmiRNA) [4]. The up-regulation of the oncomiRNA-21 has been proposed for CRC monitoring, resulting in the suppression of their tumor gene targets (PTENa, PDCD4, RECK, TPM1, SPRY2, and TIMP3) [4]. For the detection of miRNA-21, Yunlei Zhou and co-workers [118] designed a label-free electrochemical sensing approach, making use of the hairpin structure probe and hemin-G–quadruplex complex as the amplification element (Figure 5). Firstly, a 5’-thiolated hairpin DNA probe S1 was immobilized on a gold electrode modified with electrodeposited AuNP. In the presence of the target miRNA, S1 opened its hairpin structure and hybridized it with miRNA-21. Subsequently, the non-hybridized segment 3’-end of the hairpin DNA probe S1 hybridized with capture DNA S2, which was assembled on the surface of AuNPs with the aptamer DNA S3 simultaneously. Finally, the aptamer DNA S3 and hemin formed the hemin-G–quadruplex complex that was used for the quantification of miRNA-21 by an amperometric readout in the range from 5 to 5000 pM and LOD of 3.96 pM. The usefulness of the assay was demonstrated in the analysis of miRNA-21 in the total RNA extracted from gastric (BGC-823), breast (MCF-7), hepatocarcinoma (HepG2), and colon (HT-29) cancer cell lines. The results were confirmed by qPCR.

An amplification-free bioassay for the electrochemical detection of exosomal miRNA-21 isolated from a CRC cell line (SW48) and serum samples from eight patients diagnosed with CRC [119] was developed for the first time. A biotinylated capture probe was immobilized at the surface of commercial streptavidin-labeled magnetic beads trough the streptavidin–biotin interaction. After the hybridization reaction between the target and the capture probe, the miRNA-21 was separated magnetically from the total RNA extracted from exosomes. The bioconjugates were subsequently heated at 95 °C to release the captured miRNA targets, and after magnetic separation, the supernatant-containing target was adsorbed onto a screen-printed gold electrode (SPAuE) surface. The concentration of miRNA-21 was followed by DPV in the presence of the [Fe(CN)_6_]^4−/3−^ redox system from 1.0 pM to 100 nM with a LOD of 1.0 pM. The electrochemical measurements were further validated by qPCR analysis, demonstrating the viability of the bioassay for analyzing exosomal miRNA in cancer samples.

### 4.2. Electrochemical Biosensing of Protein Biomarkers of CRC

Proteins are the molecules that are responsible for a myriad of biological processes in cells and tissues, including transcription, RNA editing, proteolytic processing, and post-translational modifications. The minimum alteration in any of the processes in which they intervene may produce failures in their normal function that may end up in a type of cancer. Then, protein-based biomarkers hold potential for the screening, prediction, diagnosis, prognosis, and monitoring of CRC. Table 4 summarizes several novel and engaging formats of electrochemical biosensors based on proteic-type biomarkers that have been developed in recent years. 

One of the first electrochemical models for the prognosis of CRC was the quantification of carcinoembryonic antigen (CEA). CEA is an oncofetal glycoprotein associated with the cell surface or plasma membrane through glycosylphosphatidylinositol (GPI) [120]. The CEA level in blood in healthy individuals is 3–5 ng mL^−1^; a CEA level that exceeds 20 ng mL^-1^ strongly suggests metastatic cancer [121]. Although CEA is the most common blood biomarker for monitoring CRC after treatment [122], it is not recommended for its early detection due to its low sensitivity [32]. An amperometric biosensor was developed for the detection of CEA in serum samples from patients with colon cancer [123]. The immunosensor was constructed with SAMs of a bioconjugate based on the anti-CEA antibody and a di-thiolated aromatic compound attached to a gold electrode array placed into a microfluidic cell. The CEA antigen detection was through an HRP-labeled secondary antibody by DPV in a linear range from 0 to 200 ng mL^−1^ with a LOD of 0.02 ng mL^−1^. The concentration of CEA in real serum samples was estimated in only 10 min and compared with those from a commercial ELISA. 

Recently, the Sales M.G.F.´s group developed a new and novel electrochemical biosensor integrated into a Dye-Sensitized Solar Cell (DSSC) for the detection of CEA in urine samples [124,125,126]. The device uses a photovoltaic cell as an independent energy source that is interconnected with the biosensor to offer a new vision of affordable autonomous detection at the POC. It also includes an innovative molecularly imprinted polymer (MIP) that generated vacant positions in a semiconductor polymer matrix for the subsequent recognition of the CEA biomarker with high affinity [127]. The photoelectrochemical device was assembled on a fluorine-doped tin oxide (FTO) glass electrode with TiO_2_ sensitized with a ruthenium-based dye as a photoanode and different semiconductor polymers as counter electrodes (CEs). The analytical performance of the integrated biosensor interrogated by EIS and current-voltage (I-V) curves under specific lighting conditions was linear in concentrations ranging from 0.125 to 12.5 pg mL^−1^ with a LOD of 0.125 µg mL^−1^. The device was tested in biological urine samples doped with known concentrations of the analyte. Whereas the electrochemical response by EIS showed a LOD of 0.0832 pg mL^-1^, the I-V measurements showed a LOD of 0.091 pg mL^−1^. The increased sensitivity is related to the greater ionic strength of urine samples. 

Monitoring the electrical performance of electrochemical biosensors by self-powered systems is a new approach for tumor biomarker detection. A DSSC/biosensor was assembled with a layer of highly conductive poly (3,4-ethylenedioxythiophene) (PEDOT) for the identification of different proportions of CEA [125] and evaluated by current density-voltage (J-V) measurements in both standard CEA solutions and human urine samples from healthy individuals. The system was operated autonomously by the DSSC-generated power. The color change gradient of the electrochromic material that varied from blue to purple responded linearly with increasing concentrations of CEA in urine solutions from 10 to 100 µg mL^−1^, with an LOD of 0.14 ng mL^−1^. Based on the same principle, a DSSC-based autonomous biosensor device was assembled with polyaniline/FTO glass as a CE for the detection of CEA in urine samples [126]. The analytical performance of the system was tested in human urine samples in concentrations ranging from 0.025 to 0.75 ng mL^−1^. The use of polyaniline as MIP decreases the LOD down to 0.10 pg mL^−1^ as compared with the previous device. 

**Figure 6:**
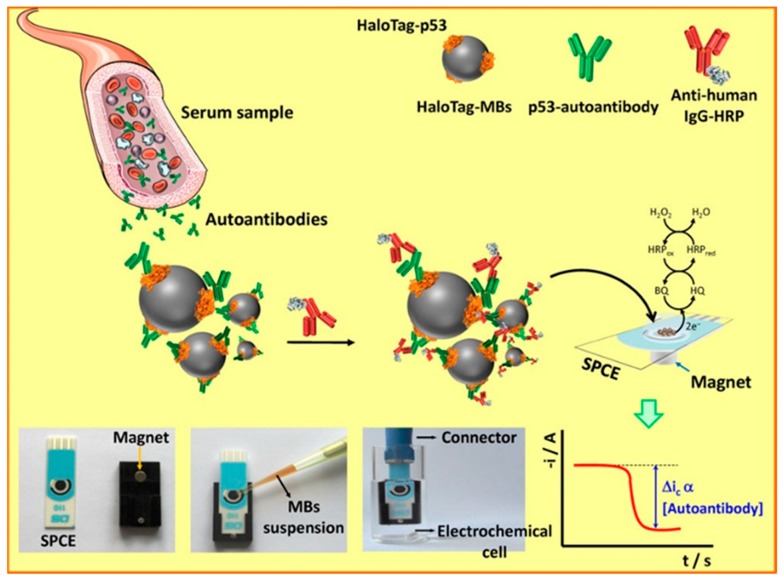
Schematic illustration of the magnetic beads (MBs) immunosensor based on the HaloTag-modified fusion protein for the detection of p53-specific autoantibodies. Reprinted from [131] with permission. Copyright © 2016, American Chemical Society.

p53 is a tumor suppressor protein called “the guardian of the genome”, which plays a crucial role in the regulation of the cell cycle, DNA repair, and programmed cell death [128]. Mutations of the p53 gene, the most common genetic alterations in human cancers, lead to the production of the mutational p53 protein, whose half-life is longer than that of the wild p53 protein [129]. Such production results in the accumulation of the mutated p53 protein together with the subsequent generation of antibodies against it. Therefore, the level of serum p53 antibodies is a potential biomarker of high stability for minimally invasive cancer malignancy screening, monitoring, and prognosis [130]. Garranzo-Asensio et al. [131] developed a biosensor based on magnetic beads (MBs) modified with covalently immobilized HaloTag fusion p53 protein as an electrochemical detection system (Figure 6). After capturing the magnetic beads bearing the immunocomplexes onto screen-printed carbon working electrodes, the bio-recognition event was monitored by the amperometric readout generated by the enzymatic reduction of H_2_O_2_ mediated by HQ, which revealed the level of p53 autoantibodies in the sample. The proposed biosensor was applied for the analysis of sera from twenty four individuals with a high risk of developing CRC and from six patients already diagnosed with ovarian and CRC, detecting low concentrations of p53 autoantibodies with an LOD of 0.34 U mL^−1^.

Similarly, a biosensor platform that combines the strength of a gold-loaded nanoporous iron oxide nanocube (Au@NPFe_2_O_3_ NC) was assembled for the early detection of p53 autoantibodies in different stages of colon cancer [132]. The Au@NPFe_2_O_3_ NC nanocomposite was functionalized with the p53 protein that acts as a capture element of the p53 autoantibodies from a sample. The resultant biocomplex was magnetically isolated and coupled with an HRP-modified secondary antibody to monitor the concentration of autoantibodies by enzymatic oxidation of 3,3’,5,5’-tetramethylbenzidine (TMB) in the presence of H_2_O_2_. The amperometric response, measured in commercial serum and serum samples from patients with colon cancer at 150 mV, was p53-specific autoantibodies concentration-dependent in a range from 0.02 to 14 U mL^−1^, with an LOD of 0.02 U mL^−1^. The use of magnetic NP facilitated the washing steps, reduced the time of analysis, and eliminated potential interferences. In addition to being fast, sensible, and cost-efficient, this method represents a new approach to detect the immune response of the body against cancer.

Similar to p53 autoantibodies, p53 protein overexpression and the presence of mutated p53 also act as tumor biomarkers of CRC [133]. Aydın M. et al. [134] developed a label-free immunosensor for the p53 antigen analysis in real human serum samples of practical clinical applicability. The specific antibody–antigen interaction was monitored by the single-frequency impedance technique (SFI) using the Bode diagram and measuring the impedance at a constant frequency value [135]. The immunosensor had a linear response from 0.02 to 4 pg mL^−1^, with an LOD of 7 fg mL^−1^, and high selectivity in the presence of proteins and drug interferences. The recovery extents between 94% and 104% demonstrated the immunosensor viability as an alternative for the analysis of the p53 antigen in the clinical routine.

**Figure 7:**
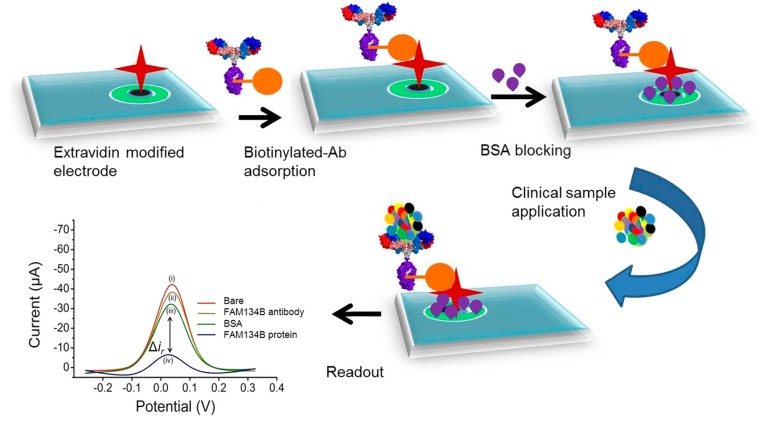
Brand-free electrochemical biosensor for the detection of the FAM134B protein in serum samples and colon cancer cell lysates. Reprinted from [138] with permission. Copyright © 2017, Springer Nature.

FAM134B is an endoplasmic reticulum resident-receptor protein that acts as a tumor suppressor. Genetic and epigenetic changes in FAM134B are related to several stages in the pathogenesis of colorectal carcinomas [136,137]. Islam et al. built the first label-free electrochemical biosensor for the quantitative detection of the FAM134B protein [138] as a biomarker in the prognosis of CRC. The biosensor relies on a biotinylated anti-FAM134B antibody anchored at the surface of an extavidin-modified SPE. The FAM134B biomarker–antibody interaction was followed by DPV in a dynamic linear range from 0.01 to 100 ng μL^−1^, with an LOD of 10 pg μL^−1^ (Figure 7). The analytical method was interrogated in the detection of FAM134B antigen in biological samples using cell lysates extracted from a panel of colon cancer cells (SW480, SW48, and HCT116) and non-neoplastic colon epithelium (FHC) cell lines [138], and the results were compared to the standard ELISA and immunostaining methods. This new label-free electrochemical biosensor provides a quick and straightforward solution in the detection of protein-based biomarkers related to CRC. 

**Figure 8:**
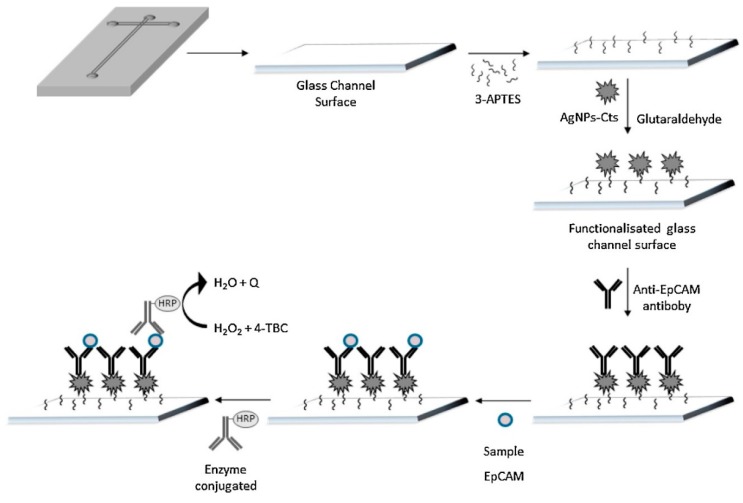
Schematic illustration of a microfluidic electrochemical immunoassay protocol for epithelial cell adhesion molecule (EpCAM) detection. Reprinted from [141] with permission. Copyright © 2015, Elsevier B.V.

The incorporation of microfluidic systems coupled to electrochemical immunosensors offers added advantages for the detection of cancer biomarkers such as high sensitivity in the analysis of complex biological fluids, portability, fast speed, and the use of small amounts of samples [139,140]. Ortega et al. [141] and Bravo et al. [142] reported electrochemical biosensors adapted to microfluidic systems for the detection of the epithelial cell adhesion molecule biomarker (EpCAM). EpCAM is a transmembrane protein expressed in several types of carcinomas of epithelial origin, including colon, prostate, liver, esophagus, breast, and lung cancer, among others [143]. EpCAM is widely used as a biomarker in sensing devices, since EpCAM is usually expressed in circulating tumor cells (CTCs) but not in healthy hematological cells [144]. Ortega et al. [141] designed a type-T format microfluidic system using glass substrates for the detection of EpCAM in biological samples (Figure 8). EpCAM antibodies were covalently attached to some chitosan-coated silver NPs (AgNPs) covering the silanized glass surface by cross-linking with glutaraldehyde. The EpCAM biomarker was linked to an HRP-labeled secondary antibody, and the enzymatic reaction in the presence of H_2_O_2_ was followed by amperometry at −0.10 V with 4-tert-butylcatechol (4-TBC) as the mediator. The microfluidic biosensor was tested with peripheral blood samples from patients with advanced metastatic CRC, whose results were dependent on the biomarker concentration with an LOD of 2.7 pg mL^−1^, which is lower than that from a commercial ELISA (13.9 pg mL^−1^). 

The microfluidic immunosensor developed by Bravo et al. [142] tested the same mentioned methodology in similar peripheral blood samples but with recombinant antibodies at polyvinyl alcohol-coated silver NPs (AgNPs-PVA). The biosensor incorporated a bispecific trifunctional monoclonal mouse antibody that recognizes specific epitopes, post-translational modifications, and conformations of the EpCAM biomarker. The three binding sites of the recombinant antibodies improved their immobilization at the AgNPs-PVA, increased the sensitivity, and reduced the LOD down to 0.8 pg mL^−1^ in a linear response range from 2 to 2000 pg mL^−1^. The microfluidic systems provide a new perspective to increase the sensitivity of conventional electrochemical models in the diagnosis and prognosis of CRC biomarkers.

A new approach for the early diagnosis of CRC in plasma samples from patients with adenomas and carcinomas used synthetic affinity peptides identified by the phage display technique [145]. Synthetic peptides were selectively bound to the leucine-rich α-2-glycoprotein 1 (LRG1) that was overexpressed in CRC patients, and the level of plasma LRG1 was related to the progression of CRC from the adenoma stage to carcinoma [146]. Four series of specific peptides that recognize the LRG1 protein were synthesized to be different in some amino acids and modified with a C-terminal cysteine and a flexible connector (-GGGGS-) to form a SAM on gold electrodes. According to CV and EIS measurements, the LRG1 BP3 peptide with the amino acid sequence of QDIMDLPDINTLGGGGSC immobilized better on the electrode and provided a more sensitive affinity for LRG1 proteins in a dynamic range of response from 0 to 0.25 μg mL^−1^, with an LOD of 0.025 μg mL^−1^. The electrochemical biosensor detected the LRG1 protein in plasma samples of patients with CRC, thus showing its ability to diagnose the adenoma–carcinoma transition in gross human plasma samples.

A bifunctional electrochemical nanobiosensor was developed for the screening and detection of chemokine (C-X-C motif) ligand 5 (CXCL5) with a natural chemokine receptor molecule (CXCR2) [147]. CXCL5 is a member of a subset of CXC chemokines that regulates cellular functions such as neutrophil trafficking and tumor angiogenesis. Therefore, the overexpression of CXCL5 may be a significant predictor of tumorigenesis and CRC prognosis and progression [148]. The nanobiosensor was manufactured on a superficially modified GCE with electroplated AuNPs and 2,2′:5′,2’’-terthiophene-3′ (p-benzoic acid) (TBA). The carboxyl groups of the pTBA layer were activated with 1-ethyl-3-(3-dimethylaminopropyl) carbodiimide (EDC)/N-hydroxysuccinimide (NHS) to link the CXCR2 receptor covalently, and the affinity was tested against CXCL5, CXCL8, and CXCL13 ligands by EIS. The results showed that the R_ct_ value increased only with increasing concentrations of CXCL5 in the sample, demonstrating the specificity of the biosensor. Another strategy was based on the union of CXCL5 with hydrazine (hyd) (CXCL5_hyd_), which acts as the electrocatalyst for the catalytic reduction of hydrogen peroxide [147,149]. The analytical performance of such a biosensor was tested by amperometry at –450 mV versus Ag/AgCl, having a linear response from 0.1 to 10 ng mL^−1^, with an LOD of 0.078 ng mL^−1^. These results are of clinical relevance, taking into account that the CXCL5 levels in serum samples from patients with CRC are from 0.20 to 5.71 ng mL^−1^ [150]. Samples of human serum and CRC cells (HT29 cells) doped with known concentrations of CXCL5_hyd_ were assessed with the electrochemical system, and the results correlated with the reference values of the white solution. Overall, this work is a step forward toward the rapid detection of CRC biomarkers in routine tests.

Ibáñez-Redín et al. reported a new biosensor platform based on homemade Ag screen-printed interdigitated electrodes (SPIDEs) modified with carbon nano-onions (CNOs) and graphene oxide (GO) films for the detection of carbohydrate antigen 19-9 (CA19-9) [151]. The flexible SPIDE electrodes were produced in two architectures (i.e., SPDE/GO-AB and SPIDE/CNO-GO-Ab) by screen-printing at Ag-coated PET substrates. The biosensor was characterized by capacitance measurements in a frequency range from 1 Hz to 1 MHz, whose response was linear in the range of 0.3 to 70 and 0.3 to 100 U mL^−1^, with an LOD of 0.12 and 0.26 U mL^−1^ for the devices with and without CNO, respectively. The decrease in sensitivity and the widening of the linear range of the CNO-based sensor is explained by the large surface area and mesoporous nature of CNO, which increased the electrical capacity and improved the analytical performance of the resultant biosensor. The device differenced whole-cell lysates of colorectal adenocarcinoma from HT29 (CA19-9 expressing) and SW620 (CA19-9 null) cell lines with 100% accuracy. This work is a boost in the implementation of non-invasive flexible biosensors in the detection of CRC biomarkers.

The overexpression of protein receptors on cancer cells also may serve as a biomarker of advanced cancer. For example, highly metastatic colon cancer cells increase the surface expression of the interleukin-13 receptor Rα2 (IL-13Rα2). The IL-13 binds to this receptor forming ligand–receptor complexes initiating signal transduction and mediating biological effects such as tumor proliferation, cell survival, cell adhesion, and metastasis. Valverde et al. reported the first electrochemical immunosensor for the determination of IL-13Rα2. The immunosensor used carboxylated magnetic beads (MBs-COOH) as solid support for the specific capture antibodies (CAb). They interacted with the target protein and with biotinylated detector antibodies (BDAb) in a sandwich-type immunoassay, followed by labeling with a streptavidin–horseradish peroxidase (strep-HRP) conjugate as a reporter. The system H_2_O_2_/hydroquinone (HQ) was employed to monitor the affinity reactions by amperometric detection at disposable SPCE. Under the optimized working conditions, the linear calibration plot for recombinant IL-13Rα2 was from 3.9 to 100 ng mL^−1^ with an LOD of 1.2 ng mL^−1^. Although the ELISA kit provides a similar LOD (0.313 ng mL^−1^), the immunosensor enables the IL-13Rα2 determination in a much shorter time (1 h 15 min versus 4 h 40 min, once MBs-CAb and plate-CAb were prepared, respectively). 

Furthermore, the immunosensor requires only portable and cost-effective instrumentation, making it more easily automated and miniaturized, which is ideal for decentralized settings. The immunosensor determined the target receptor in raw lysates from both CRC and intact cells. The results showed a more abundant expression of the IL-13Rα2 in the highly metastatic cells (SW620 and KM12SM) in comparison to their isogenic cell pairs (SW480 and KM12C). Moreover, the immunosensor for KM12SM provides an amperometric signal 1.5-fold higher than that for KM12C, demonstrating its potential to discriminate metastatic properties in intact cells through IL-13Rα2 expression. The high analytical performance exhibited by this immunosensor, along with its rapid and straightforward operation, shows its potential for routine metastatic CRC detection at the POC [152].

**Figure 9:**
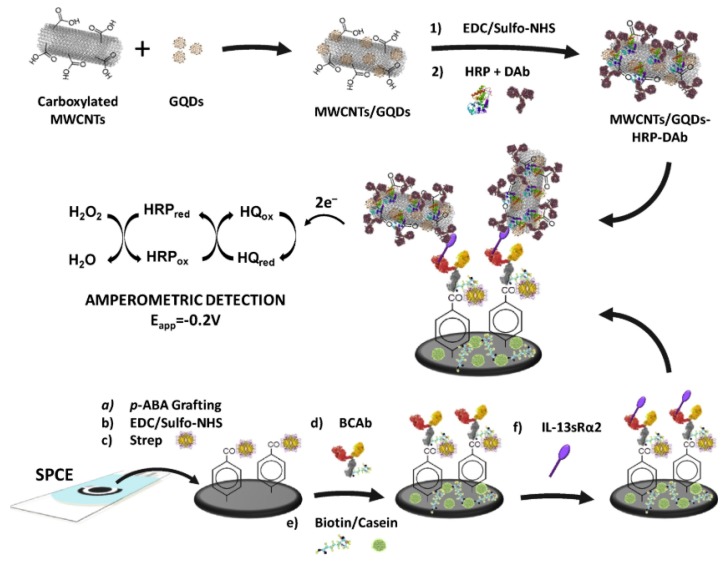
Scheme of the different steps involved in the development of a sandwich-type amperometric immunosensor for interleukin-13 receptor Rα2 (IL-13Rα2) detection. Reprinted from [153] with permission. Copyright © 2019, Elsevier B.V.

After the mentioned pioneering work, Prof. Pingarrón’s research group developed the first integrated electrochemical immunosensor for monitoring IL-13Rα2. The biosensor consisted of p-aminobenzoic acid (pABA) grafted at an SPCE, which was activated via the EDC/Sulfo-NHS chemistry to covalently immobilize streptavidin and link a biotinylated specific capture antibody. A nanohybrid based on MWCNTs/graphene quantum dots (GQDs) conjugated to HRP and a detection antibody (DAb) was implemented as a label in a sandwich-type immunoassay (Figure 9). The combined properties of MWCNTs and GQDs promoted the electron transfer between the redox probe and the electrode surface for the signal amplification. Under optimized conditions, the cathodic current of the biosensor, measured by amperometry with the H_2_O_2_/HQ system, responded in an IL-13Rα2 concentration-dependent manner from 2.7 to 100 ng mL^−1^ with improved sensitivity and an LOD of 0.8 ng mL^−1^. The LOD was slightly lower than that from an immunosensor based on MBs, and the time of assay also was less than 2 h. Concentrations of IL-13Rα2 from colon cancer cell lysates (SW480, SW620, KM12C, KM12SM) and paraffin-embedded colorectal tissues were in agreement with those reported for the same cells using the MBs-based immunosensor. Besides, more significant contents of IL-13Rα2 were found in extracts from CRC tissues as compared to healthy tissues. This immunosensor exhibits attractive analytical characteristics in terms of selectivity, sensitivity, reproducibility, and stability and the possibility not only of using a small amount of cells lysates and extracts from paraffin-embedded tumor tissues but also simple protocols with minimal sample treatments [153]. 

Similarly to the immunosensor developed for the determination of IL-13Rα2 [153], Valverde et al. proposed an immunosensor to determine the receptor activator of nuclear factor-κB ligand (RANKL) [154]. The ligand RANK was recently found in the tumor microenvironment contributing to cancer progression [154,155]. The authors integrated AuNP-MWCNT-containing nanohybrids as nanocarriers of multiple detector antibodies and HRP (MWCNTs/AuNP-HRP-DAb) for signal amplification in conjunction with biotinylated capture antibodies immobilized onto an SPCE/pABA/strep platform. The amperometric readout with the H_2_O_2_/HQ system showed a dynamic linear range from 10.4 to 1000 pg mL^−^^1^ with an LOD of 3.1 pg mL^−^^1^. The nanohybrid MWCNTs/AuNPs achieved a higher sensitivity as compared to the previous nanocarrier (MWCNTs/CQDs). RANKL levels in serum specimens from patients diagnosed with CRC in stages III and IV found with the biosensor were comparable with those obtained by ELISA, thereby demonstrating the reliability of the new device.

**Figure 10:**
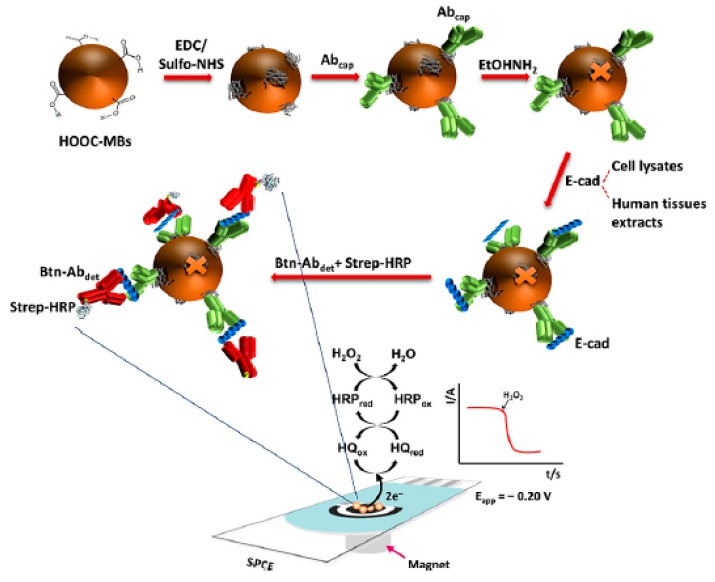
Scheme of the fabrication procedure and amperometric transduction system based on a sandwich immunosensor format developed for E-cadherin (E-cad) determination. Reprinted from [160] with permission. Copyright © 2019, Wiley-VCH Verlag GmbH & Co. KGaA, Weinheim.

Cadherins are a family of transmembrane glycoproteins that mediate calcium-dependent cell–cell adhesion. These glycoproteins form cadherin–catenin complexes that provide normal cell–cell adhesion and maintain homeostasis and stability in epithelial tissues [156]. The down-regulation of E-cadherin (E-cad) is associated with tumor progression, the loss of differentiation, invasion, and metastasis, so it is considered a relevant biomarker of colon cancer prognosis [157]. In contrast, in CRC, Cadherin-17 (CDH17) is overexpressed and has been suggested as a useful biomarker for identifying adenocarcinomas in a metastatic stage [158,159]. Muñoz-San Martín et al. reported the first immunosensor for monitoring E-cad, as shown in Figure 10. The platform was based on a sandwich configuration onto carboxylated magnetic microcarriers, using two specific antibodies against E-cad protein: a capture antibody (E-cad Ab_cap_) and a secondary biotinylated antibody (Btn-Ab_det_) labeled with streptavidin–HRP conjugate (HRP-Strep). The E-cad Ab_cap_ was immobilized on the MBs via EDC/Sulfo-NHS chemistry, and the target protein was sandwiched with the Btn-Ab_det_/HRP-strep conjugate. The immunocomplexes attached to the MBs were captured magnetically onto SPCE, and the amperometric response with the H_2_O_2_/HQ system was E-cad concentration-dependent from 0.5 to 25 ng mL^−1^ with an LOD of 0.16 ng mL^−1^. The immunosensor was interrogated in both CRC lysates (SW480, SW620, KM12C, and KM12SM) and tumoral tissues. In metastatic cells (SW620 and KM12SM) and tumor tissues, the content of E-cad was significantly lower as compared with the non-metastatic cells and healthy tissues. Moreover, the sensing platform is compatible with miniaturization and integration into multiplexed devices, thus holding the potential to meet the current clinical demands in CRC diagnosis and prognosis [160]. 

**Figure 11:**
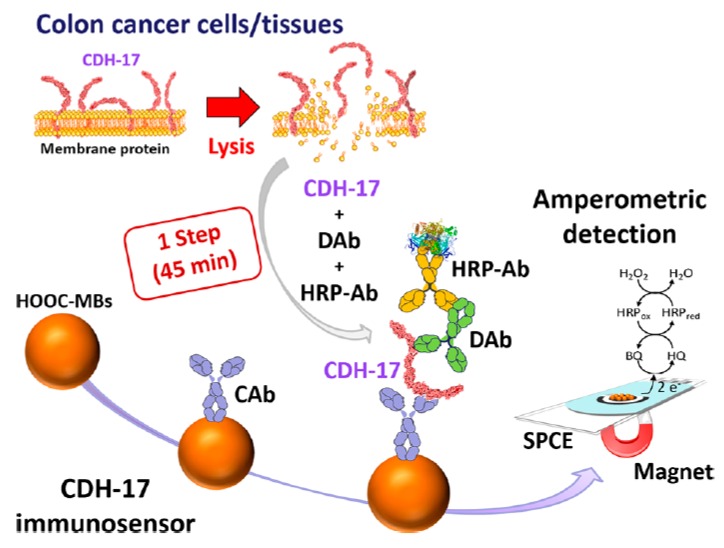
Scheme of an immunosensor developed for the determination of Cadherin-17 (CDH-17). Reprinted from [161] with permission. Copyright © 2018, American Chemical Society.

Valverde et al. developed the first biosensor for the determination of CDH17. The strategy for detecting the target protein involved a sandwich-type immunoassay assembled onto magnetic microparticles by using a capture antibody (CAb) anti-CDH17, a detection antibody (DAb), and an anti-immunoglobulin G (IgG)–HRP conjugate as an enzymatic label (Figure 11). The biosensor comprised the CAb attached to the MBs-COOH surface via EDC/Sulfo-NHS reaction, the formation of immunocomplexes between Cab, CDH17, and DAb-anti-IgG-HRP; and amperometric transduction at SPCE with the H_2_O_2_/HQ system. The biosensor exhibited a linear dependence of the measured cathodic current with the CDH17 concentration in the range of 4.8 to 1000 ng mL^−1^ and an LOD of 1.43 ng mL^−1^. The content of CDH17 was determined in raw cell lysates (SW480, SW620, KM12C, and KM12SM) and tumoral tissues, and the overexpression of CDH17 was verified in metastatic cells and tissues in only 45 min, with low amounts of samples. The simplicity, affordability, speed, and portability of the device make it potentially useful as a POC clinical tool [161]. 

Narajan et al. fabricated a simple label-free immunosensor for monitoring the peptide endothelin 1 (ET-1) colon cancer biomarker. The ET-1 is a vasoconstrictor peptide chain of 21 amino acids that can be found in elevated levels in the blood serum of CRC patients. Modification of the working gold electrode with an SAM of 11-mercaptoundecanoic acid (11-MUA) and the covalent immobilization of a monoclonal anti-ET-1 antibody via EDC/NHS chemistry were used to prepare the biosensing platform. The electrochemical detection of molecular binding between the anti-ET-1 and ET-1 peptide was conducted by EIS, using 5 mM [Fe(CN)_6_]^3^^−^^/4^^−^ in PBS buffer (pH 7.4) as a redox probe. The R_ct_ increased as the antigen–antibody complexes that hindered the electron transport were effectively formed on the modified electrode in a concentration-dependent way. The impedimetric response was linear in a concentration ranging from 2 to 100 pg mL^−1^ with an LOD of 0.36 pg mL^−1^. Furthermore, the immunosensor was also characterized by the surface plasmon resonance technique and applied to a serum sample analysis of CRC patients. This label-free immunosensor was found to be more sensitive, less time consuming, and exhibited better LOD than the ELISA kit [162].

**Figure 12:**
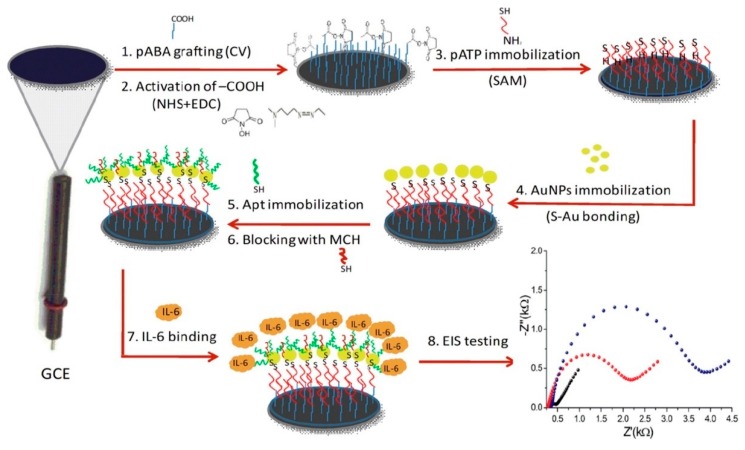
Diagram of aptasensor preparation for the detection of interleukin-6. Reprinted from [164] with permission. Copyright © 2019, Elsevier B.V.

New elements of biological recognition have been recently incorporated into the manufacture of tumor biomarker biosensors. An innovative format has been based on aptamers (Apt), which can be oligonucleotides with an extraordinarily high and specific affinity for the target [163]. Tertis et al. [164] proposed a particular platform consisting of nanocomposites for the detection of interleukin-6 (IL-6) in human blood. The electrochemical biosensor was based on the modification of GCE with p-aminobenzoic acid, p-aminothiophenol, and AuNP, as shown in Figure 12. The thiolated aptamer, specific for IL-6, was immobilized on the modified-GCE through sulfur–gold bonds, offering a stable and reproducible platform for the target-protein capture. EIS evaluated variations of Rct with different concentrations of IL-6 in the presence of [Fe(CN)_6_]^3^^−/4^^−^, whose response was linear from 0.005 to 100 ng mL^−1^ with a quantification limit (LOQ) and LOD of 5 and 1.6 pg mL^−1^, respectively. The LOD is considered of clinical relevance, since serum IL-6 levels are in the range of 4 to 6 pg mL^−1^ [165]. The aptasensor was evaluated with real samples of patients suffering from CRC and validated with a chemiluminescence immunoassay (CLIA). The approach was shown to be amenable for the miniaturization, multiplexing, design, and manufacturing of devices at the POC.

### 4.3. Electrochemical Biosensing of CRC-Associated Cells

Circulating tumor cells (CTCs) are whole single cells and cell clusters released from tumors into the blood [166]. The detection and quantification of CTCs are of great interest in monitoring the cancer stage [167], predicting patient prognosis [168], and evaluating treatment [169]. Subsequent analysis of the genomic and proteomic markers associated with the CTCs is critical for improving the early detection or predicting the response to therapy in CRC [170], making this approximation a potential alternative to invasive biopsies [171]. Different studies have been published for the detection of CTCs in CRC, as listed in Table 5. Maltez-da Costa et al. presented a fast and straightforward strategy for the quantification of the human colon adenocarcinoma cell line (Caco2) using NP. The Caco2 cells overexpress EpCAM and CEA, which were electrochemically detected with different antibodies conjugated to AuNPs in a sandwich with some antibody-modified superparamagnetic microparticles. The amperometric response against Caco2 cells was followed by amperometry through the Hydrogen Evolution Reaction (HER) electrocatalyzed by AuNP labels, in the presence of other circulating cells (monocytes THP-1) that could interfere in real blood samples. The response was linear from 1 × 10^4^ to 5 × 10^4^ cells for the anti-EpCAM-AuNP and the anti-CEA-AuNP with an LOD of 8.34 × 10^3^ cells and 2.2 × 10^2^ cells, respectively. Remarkably, both MBs and AuNPs were not competing for the same antigen in the cell surface, maximizing the number of AuNPs attached to that. Furthermore, by modifying the capture and detection conjugates with specific antibodies, the platform could detect other CTCs [172]. 

Some cytosensors have been developed for the detection of the epithelial cancer cell line HCT116, epithelial-like cancer cell line HT29, and CT26 mouse colon carcinoma, which express CEA glycoproteins at their cell surface. For example, Raji et al. modified gold electrodes with a SAM of 11-mercaptoundecanoic acid (11-MUA) and immobilized the NH_2_-KCHA10a aptamer via 1-ethyl-3-(3-dimethylaminopropyl) carbodiimide (EDC)/N-hydroxysuccinimide (NHS). The CRC cells were detected by CV, with 1 mM [Fe(CN)_6_]^3−^ as the redox probe, whose response was linear from 1 to 100 cells mL^−1^ with an LOD of 7 cells mL^−1^. Besides, the selectivity of the aptasensor was evaluated in the presence of the Hep-2 epithelial cell, demonstrating high performance [173].

Jing et al. developed a cytosensor for HCT116 cell capturing based on a nanostructured biosensing interface with hyaluronate-functionalized graphene (HG). The HCT116 cell surface is rich in CD44 hyaluronic acid (HA) receptor and can be detected with the cytosensing platform by the HA–CD44 protein interaction. The nanobiointerface was prepared by coupling amine-functionalized graphene oxide (NH_2_/GO) HA via EDC/NHS chemistry and the subsequent coating of the GCE with the nanocomposite. The cytosensor signal response, interrogated by both CV and EIS in 2 mM of [Fe(CN)_6_]^3^^−/4^^−^, was linear from 5.0 × 10^2^ to 5.0 × 10^6^ cells mL^−1^, with an LOD of 100 cells mL^−1^ and with acceptable precision and fabrication reproducibility [174]. 

**Figure 13:**
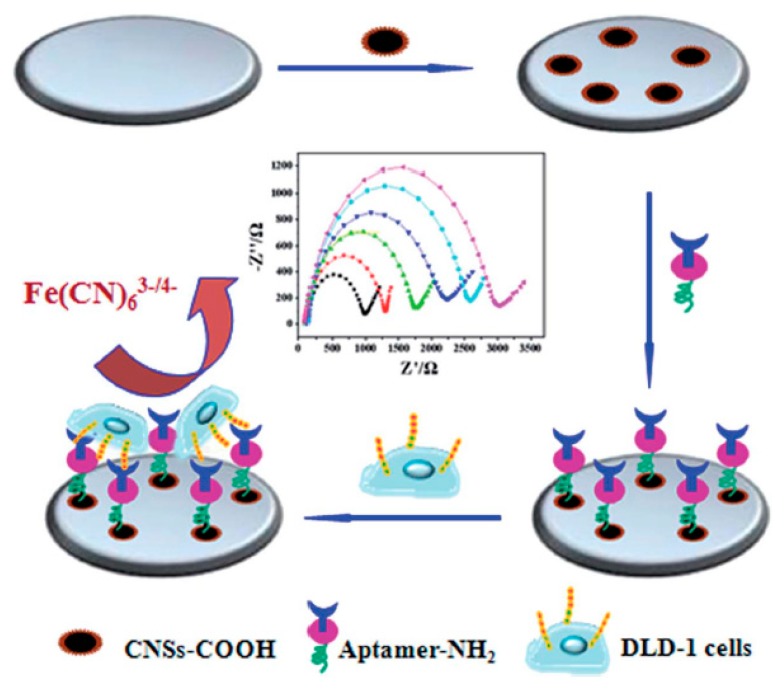
Schematic representation of carboxylated carbon nanospheres (CNSs)-based aptasensing strategy for human cancer DLD-1 cell detection by the EIS technique. Reprinted from [177] with permission. Copyright © 2014, Royal Society of Chemistry.

Mucin-1 (MUC-1) is an overexpressed cell surface glycoprotein that is present in the tumor cells of CRC and is confirmed as a biomarker for its early diagnosis [175,176]. Cao et al. established an aptasensor with specificity against MUC-1. The sensing platform was assembled by drop-casting carboxylated carbon nanospheres (CNSs-COOH) on the surface of a GCE and subsequent NH_2_-MUC-1 aptamer conjugation via EDC/NHS. The aptamer recognizes the MUC-1 glycoprotein on the cellular membrane of human cancer colon DLD-1 cells, and their attachment onto the sensing platform was monitored by EIS using 10 mM [Fe(CN)_6_]^3−/4−^ as the redox probe (see Figure 13). At optimized conditions, the aptasensor responded in a concentration ranging from 1.25 × 10^2^ to 1.25 × 10^6^ cells mL^−1^ with an LOD of 40 cells mL^−1^. This aptasensor can detect DLD-1 cells in the presence of human astrocytes 1800 cells, thus demonstrating its high specificity [177]. 

**Figure 14:**
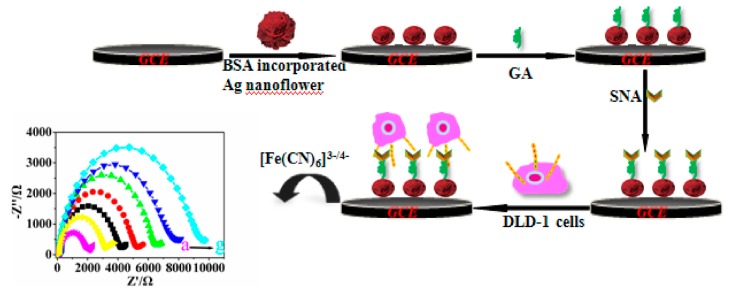
Development of an EIS cytosensor based on BSA-incorporated Ag nanoflowers for DLD-1 cell detection. Reprinted from [180] with permission. Copyright © 2015, Elsevier B.V.

Human colon cancer cells overexpress highly sialylated glycans at the cell surface, which are attached to proteins and lipids [178]. Furthermore, sialylation levels are altered during cancer progression [179]. To detect sialic acid (SA) on the DLD-1 cell surface, Cao and co-workers developed a label-free cytosensor based on a protein–inorganic nanomaterial, which incorporates Ag nanoflowers in Bovine Serum Albumin (BSA) with three-dimensional porous architectures as sensing interfaces (Figure 14). The BSA-incorporated Ag nanoflowers were drop-cast on a GCE surface and conjugated to the R-type lectin *Sambucus nigra* agglutinin (SNA) via glutaraldehyde cross-linking. The SNA had a highly specific binding affinity with SA and allowed capturing DLD-1 cells by specific binding with these groups. The flower-like nanostructure was porous, and a large number of self-assembled Ag NP increased the effective surface area for bioreceptor attachment and promoted electron transfer. The protein layer of BSA is a multifunctional platform, allowing immobilization of the bioreceptor SNA and blocking nonspecific interaction sites. Furthermore, the nano-biointerface showed high biocompatibility as demonstrated by the 3-(4,5-Dimethylthriazol-2-yl)-2,5-diphenyl tetrazolium bromide (MTT) cytotoxicity assay. The cytosensor response, which was evaluated by EIS with 10 mM [Fe(CN)_6_]^3^^−/4^^−^ as the electroactive redox probe, was linear from 1.35 × 10^2^ to 1.35 × 10^7^ cells mL^−1^ with an LOD of 40 cells mL^−1^. Therefore, the cytosensor enabled the detection of SA-positive tumor cells in the presence of human embryonic kidney 293 (HEK293) and human astrocyte 1800 cells, thus demonstrating potential for the early diagnosis of human colon cancer [180]. 

**Figure 15:**
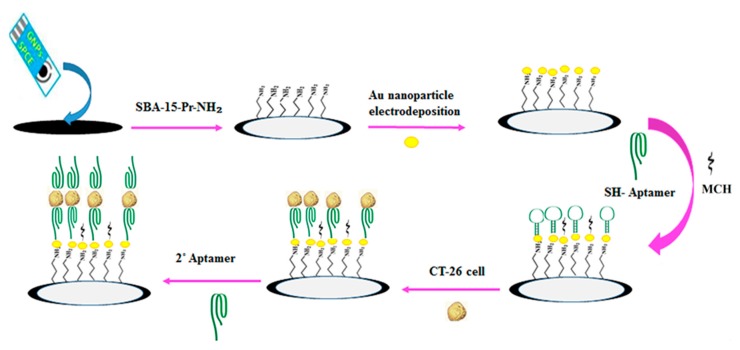
Different steps of an aptasensor construction based on a thiol terminated aptamer (5TR1) for the detection of CT26 cancer cells. Adapted from [181] with permission. Copyright © 2017, Elsevier B.V.

Hashkavayi et al. manufactured an electrochemical aptasensor that incorporated nanomaterials in the biosensing platform to increase the electrical conductivity and improve the rate of electron transfer between the electrode and a redox probe (Figure 15). By modifying mesoporous silica SBA-15 with 3-aminopropyltriethoxysilane (APTES), they obtained an amine-functionalized nanomaterial, namely SBA-15-pr-NH_2_, which was drop-cast at an SPCE with further electroplating of AuNPs via amperometry. Next, a DNA aptamer with 5′-thiol modification (5TR1) was self-assembled onto AuNPs/SBA-15-pr-NH_2_/SPCE surface and used to efficiently capture mouse colon adenocarcinoma CT26 cells in between a secondary aptamer in a sandwich-type format. The electrochemical response of the aptasensor, measured by both CV and EIS with 10 mM [Fe(CN)_6_]^3−/4−^ as the redox probe, was linear from 10–1.0 × 10^5^ and 6.0 × 10^6^ cells mL^−1^ with an LOD of 2 cells mL^−1^. Furthermore, the aptamer-CT26 cell-binding constant value was estimated to be 2.9 × 10^6^ M, which indicates a high affinity between them. This label-free aptasensor exhibited some other advantages such as simplicity, rapidity, high selectivity, and sensitivity toward the detection of CT26 cancer cells [181]. 

The functionalization of electrode surfaces with silicon-based nanomaterials has been explored to enhance the cytosensors’ electrochemical performance. Soleymani et al. applied functionalized fibrous nano-silica KCC-1 to detect CRC cells by folate (FA)–folate receptor (FR) interactions. FR is an overexpressed protein at the HT29 cancer cell surface that strongly and specifically interacts with FA. KCC-1 nanomaterial was synthesized via the hydrothermal method and amine-functionalized with APTES. The COOH group of FA was activated with EDC/NHS and linked to KCC-1-NH_2_. The KCC-1-NH_2_-FA nanomaterial was anchored on a GCE surface by amperometry at a negative potential of −0.24 V for 500 s. Whereas the electrochemical cytosensor was characterized by SWV, DPV, and EIS, in the presence of the [Fe(CN)_6_]^3−/4−^ redox probe, the DPV/SWV enabled the detection of HT29 cancer cells. The electrochemical signals decreased with the increase in cell concentration in the linear range from 50 to 1.2 × 10^4^ cells mL^−1^, with a lower limit of quantification (LLOQ) of 50 cells mL^−1^. The developed device provides excellent specificity and sensitivity, which is ideal for POC applications of clinical use [182].

The direct detection of metabolites related to cancer cell activity has been also developed for the disease prognosis. Majidi et al. engineered an ultrasensitive label-free aptasensor for the rapid screening of the essential amino acid L-tryptophan (Trp). The Trp consumption rate analysis was implemented as a prognostic marker in different cancer cell lines because it is higher in HT29 cells as compared with other cancer cell lines, including HepG2 (hepatocarcinoma) and 1321NI (astrocytoma). The aptasensor was prepared by the decoration of a gold electrode with (MWCNTs-COOH) and physical adsorption of the aptamer. The folding of aptamer molecules occurs in the presence of Trp at the modified electrode surface, enabling the concentration of the analyte for its oxidation at a fixed potential. Trp was quantified by constant current-potentiometric stripping analysis (CC-PSA), amperometry, and DPV. The signal response was Trp concentration-dependent in the ranges of 0.0001 to 10 and 10 to 300 mM, with an LOD of 64 pM. Furthermore, the aptasensor detected Trp in biological matrices such as human blood serum, saliva, and urine, whose values were in agreement with those from the HPLC method [183].

Microfabricated electrodes have assessed the biosensing of cancer cell activity. Ragones and collaborators developed a novel disposable 3D printed electrochemical sensor for the rapid detection of the ALP biomarker, which was secreted from colon cancer cell lines. The biosensing system consisted of the arrangement of nine chips made of a biocompatible substrate with two gold electrodes (working and counter) and an Ag/AgCl quasi-reference electrode. A 3D negative mold was designed and printed, polydimethylsiloxane (PDMS) was poured into the mold, and the sample was cured at 55 °C for 2 h. Carbon electrodes were formed on the chip surface by filling the substrate trenches, which were patterns, with a conductive PDMS–graphite mix and curing them at 80 °C for at least 8 h. The surfaces of carbon electrodes were gold-coated by sputtering, and the reference electrode was made by electroplating and anodization. A holder made from the thermoplastic polymer acrylonitrile butadiene styrene (ABS) was used to embed the chip. The electroactivity of the working electrode was verified by CV using 10 mM [Fe(CN)_6_]^−3/−4^ as the redox probe, and the secreted ALP enzyme levels were measured to demonstrate direct *in vitro* cell monitoring in HT29, HCT116, and Colo320 cell lines. The enzymatic activity was measured upon the addition of the substrate p-aminophenyl phosphate (pAPP) to the cell culture, which undergoes dephosphorylation, yielding the electroactive product p-aminophenol (pAP). Subsequently, pAP was oxidized to iminoquinone on the working electrode and then monitored by amperometry. The biosensor response was linear from 0.25 to 10 µg mL^−^^1^, and ALP cell levels were of 3.027, 1.774, and 1.390 µg mL^−^^1^ for Colo320, HCT116, and HT29, respectively [184].

### 4.4. Electrochemical Multibiosensing of CRC-Associated Biomarkers

CRC is a heterogeneous disease at the molecular level that involves genomic and transcriptomic changes [185,186], as well as post-translational modifications [4]. Therefore, the use of a single biomarker for diagnosis and/or prognosis lacks sufficient specificity for reliable and accurate patient monitoring. Hence, the use of a panel of biomarkers is more appropriate to improve the accuracy of diagnosis, prognosis, and cancer outcomes, as demonstrated by the FDA-approved tests that are currently directed toward this modality [29,37,38,39,44,187]. Furthermore, multi-biomarker analysis is required, because some of the molecular events that occur around the initiation and progression of a tumor can also be triggered in other pathologies, so the determination of a single tumor marker leads to an inaccurate and erroneous diagnosis [188,189].

**Figure 16:**
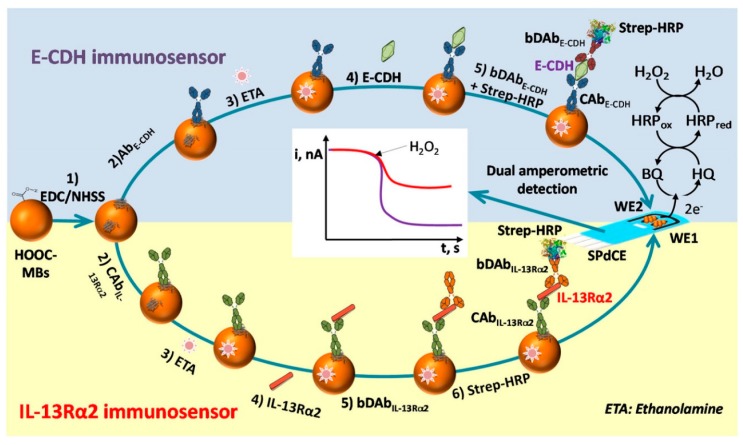
Scheme of a dual immunoassay for the simultaneous detection of IL-13Rα2 and E-CDH. Reprinted from [190] with permission. Copyright © 2019, Wiley-VCH Verlag GmbH & Co. KGaA, Weinheim.

Despite the increasing interest in monitoring a panel of biomarkers for the accurate diagnosis and monitoring of CRC, little progress in the multiplexed electrochemical biosensing has been reported so far. In this regard, Prof. Pingarrón’s research group has demonstrated the potential of electrochemical biosensors in the simultaneous determination of two CRC-associated biomarkers in a single run, i.e., the interleukin-13 receptor α2 (IL-13Rα2) and cadherin (E-CDH or CDH-17), which are of relevance in metastasis processes. By coupling previously established single approaches [152,153,160], two dual sandwich-type immunoassays were assembled to simultaneously determine IL-13Rα2 and E-CDH in the first bioassay and the IL-13Rα2 and CDH-17 in the second one. In the first dual bioassay [190], each target protein was sandwiched between the specific capture antibodies covalently immobilized onto MBs-COOH and biotinylated detector antibodies labeled with streptavidin–HRP conjugates. After that, each sandwich immunocomplex was magnetically captured on the corresponding working electrode of screen-printed dual carbon electrodes (SPdCE), as shown in Figure 16. This double immunoassay was applied in the analysis of three kinds of biological samples, i.e., lysates from two isogenic pairs of colorectal cell lines with different metastatic potential (SW480/SW620 and KM12C/KM12SM), extracts from solid tumor and adjacent healthy tissues from four patients, and serum from four patients diagnosed with CRC at different stages and four healthy individuals. 

The second dual biosensor involved the use of nanocarriers as an amplification strategy [191]. Each sandwich assay uses the corresponding specific antibodies against IL-13Rα2 and CDH-17 covalently immobilized onto the SPCE electrochemically grafted with p-aminobenzoic acid. A hybrid composed of GQDs and MWCNTs acted as a nanocarrier of both detection antibodies and HRP. Unlike the first sandwich-type immunoassay format, this dual immunosensor allowed the determination of both biomarkers not only in lysates from CRC cell lines (SW480/SW620 and KM12C/KM12SM) and paraffin-embedded tissue but in whole cells without previous lysis or permeabilization. A dual amperometric readout of the catalytic current produced upon H_2_O_2_ addition using HQ as a redox mediator was employed in both double immunosensor formats to monitor each target biomarker concentration providing LODs at the level of ng mL^−1^ in all cases. For the first dual immunoassay, the linear range was from 3.4 to 100 ng mL^−1^ with an LOD of 1.03 ng mL^−1^ for IL-13Rα2, and from 0.9 to 25 ng mL^−1^ with an LOD of 0.26 ng mL^−1^ for E-CDH, respectively. In the second case, the linear range was from 4.92 to 100 ng mL^−1^ with an LOD of 1.44 ng mL^−1^ for IL-13Rα2, and from 0.11 to 10 ng mL^−1^ with an LOD of 0.03 ng mL^−1^ for CDH-17, respectively. 

**Figure 17:**
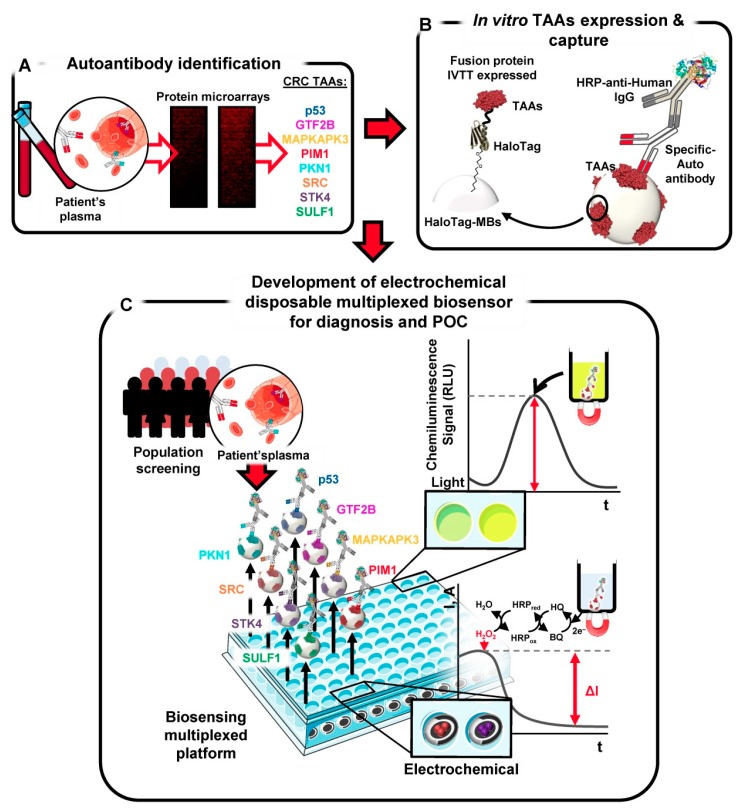
Schematic design of the strategy for the simultaneous electrochemical determination of a panel of autoantibodies composed of eight tumor-associated antigens (TAAs). Reprinted from [48] with permission. Copyright © 2020, Ivyspring International Publisher.

Recently, a novel multiplexed electrochemical biosensor for the simultaneous detection of CRC-specific autoantibodies in plasma samples was developed by Garranzo-Asensio et al. [48], similarly to that previously reported for a single tumor-associated antigen (TAA) (p53) [131]. The multiplexed immunosensor used HaloTag fusion proteins self-assembled on magnetic microparticles and amperometric detection at disposable SPCEs in the presence of the H_2_O_2_/HQ system (Figure 17). The authors tested the immunosensor by detecting a new panel of autoantibodies against eight TAAs (GTF2B, MAPKAPK3, PIM1, PKN1, SRC, STK4, SULF1, and p53) in asymptomatic plasma samples, which were samples from premalignant individuals, CRC patients, and samples from breast and lung cancer patients. By detecting eight autoantibodies in a multiplexed manner, the immunosensor discriminated asymptomatic individuals and CRC patients, while premalignant individuals reacted only to 4 TAA. The panel of autoantibodies was specific for CRC, indicating its great potential as a POC device for serum or plasma analysis of CRC in early stages. Chemiluminescence tests showed that many CRC patients reacted to all TAAs. Besides, the HaloTag technology allowed covalent interaction with magnetic beads, thus improving the solubility of fusion proteins, yields, and sensitivity; and minimizing matrix effects [48]. The device is an innovative and competitive alternative for the early and reliable diagnosis of CRC.

## 5. Current Challenges and Future Perspectives in Electrochemical Biosensing of CRC-Associated Biomarkers 

We have focused so far on describing the enormous potential of electrochemical biosensors in tumor biomarker determination, including molecules of different natures and diverse roles in disease diagnosis, prognosis, and therapeutic response. Most of the examples reported in the literature include wild-type genes, point mutations in genes, microRNA, proteins, peptides, and cells as targets of CRC. These outstanding contributions demonstrate the potential of electrochemical biosensors for CRC biomarkers monitoring to be incorporated as POC testing soon. The high versatility of electrochemical biosensors comes from coupling different bioreceptors with superior nanomaterials and electrode surfaces, implementing a variety of amplification strategies and bioformats, and taking advantage of the simplicity of the electrochemical detection techniques. Such outstanding features ensure their specificity, selectivity, sensitivity, and applicability in real scenarios. 

Although biomarkers are one of the most valuable and promising tools to get a complete cancer monitoring, from the screening step to the diagnosis and follow-up after treatment, the truth reliability of a biomarker is given mainly by its specificity, sensibility, and its correlation with the tumoral progression or regression [188]. Yet, at this point, no biomarker that meets the principles mentioned above has been identified so far; thus, there is not yet an ‘ideal biomarker’ for the specific detection of CRC [189]. This fact is closely related to the complex molecular events that are involved at a genetic, regulatory, and functional level that lead to imprecision, misdiagnosis, and cross-reactivity. Furthermore, in most cases, the abnormality of a biomarker is only expressed in some stages of the pathology, so its simultaneous use as diagnostic, prognostic, and therapy response evaluation of the biomarker is invalid. What is clear now is that the multitarget testing of carefully selected biomarkers (which can include multiple biomarkers at different molecular levels or clinical ranges) is imperative in the early detection, differential diagnosis, prognosis, and response to therapy; it may revolutionize the routine clinical practice of CRC monitoring. 

The available tests approved by the FDA for cancer monitoring and follow up are a good example of the multiplexed determination of biomarkers in biological fluids, in which the limitation of accessibility is overcome. However, the selectivity and sensitivity are not yet comparable with those from the primary screening tools. Yet, a more significant test performance could be achieved by detecting multiple-level biomarkers.

The pioneering results regarding multiparameter biosensing suggest that under this modality, cross-talking is controlled, and the results are comparable to those from the single determination of the corresponding biomarkers. However, subsequent studies in the simultaneous determination of biomarkers of different molecular levels are required to increase the diagnostic accuracy. Furthermore, studies that focus on such would emphasize the advantages of electrochemical biosensors as compared to gold standard methods such as qPCR and ELISA in the simultaneous determination of biomarkers at different molecular levels, with not only short analysis time and simple protocols but a minimal amount of sample requirements.

On the other hand, it has been seen that the determination of biomarkers in both liquid and solid biopsies is compatible with the electrochemical instrumentation. However, the discovery of free tumor cells, cell-free nucleic acids (cfDNA and cfRNA, among others) and proteins promote the use of circulating biomarkers as excellent candidates to the transition toward liquid biopsies, minimally invasive testing, and personalized medicine. Overall, the unique features offered by electrochemical biosensors such as their versatility, fast response, accurate quantification, and amenability for multiplexing and miniaturization position them at the forefront of cancer diagnosis and monitoring research. Although progress in this field requires a joined effort among molecular biologists, nanotechnologists, electrochemists, engineers, and clinical doctors, among others, they hold considerable promise for CRC biomarkers monitoring at the POC.

## Figures and Tables

**Table 1:** Tumor biomarker-based diagnosis and monitoring of CRC applied in routine clinical practice.

Clinical Purpose	Type of Biomarker	Tumor Marker	Specimen Type	Commercially Available Test	Detection Method	Ref.
Evaluation of the risk/probability of a patient having CRC	Messenger RNA (mRNA)	ANXA3, CLEC4D, LMNB1, PRRG4, TNFAIP6, VNN1, IL2RB	Blood	ColonSentry®	qPCR	[25,26,27]
Screening for average-risk adults	Mutant and methylated DNA, and FIT	-	Stool	^a^ Cologuard^TM^	For DNA markers: qPCR For Hemoglobine: ELISA	[28,29]
	Methylated DNA	SEPT9	Blood	^b^ Epi proColon®	qPCR	[25,30,31]
Diagnostic	Glycoprotein	^c^ TAG-72	Serum	NA	-	[32]
	Conjugated polypeptide	TPS	Serum	NA	-	[32]
Prognosis	Tetrasaccharide	^d^ CA-19.9	Serum	NA	Immunoassay	[32,33]
Evaluation of recurrence after tumor resection	Glycoprotein	^e^ CEA	Serum	NA	Immunoassay	[25,32,34,35]
Therapeutic-response (5-Fluorouracil-based chemotherapy)	Conjugated polypeptide	TPS	Serum	NA	-	[32]
	Glycoprotein	^e^ CEA	Serum	NA	Immunoasay	[25,32,34,35]
Therapeutic-response (cetuximab and panitumumab-based therapy)	DNA	Mutations in KRAS gene	DNA samples extracted from FFPE tumor tissue	^f^ Therascreen KRAS RGQ PCR Kit	qPCR	[36]
		Mutations in codons 12 and 13 of the KRAS gene	DNA samples extracted from FFPE tumor tissue	^g^ Cobas KRAS Mutation Test	qPCR	[37]
		For cetuximab: KRAS wild-type (absence of mutations in codons 12 and 13) For panitumumab: KRAS (exons 2, 3, and 4) and NRAS (exons 2, 3, and 4)	DNA samples extracted from FFPE tumor tissue	^h^ FoundationOne CDx	-	[38]
Therapeutic-response (panitumumab-based therapy)	DNA	Mutations from exons 2, 3, and 4 of both KRAS and NRAS gen	DNA samples extracted from FFPE tumor tissue	^i^ Praxis Extended RAS Panel	qPCR	[39]
Follow-up of metastatic patients	Circulating tumor cell	Epithelial cells	Blood	^j^ CellSearch™	Fluorescence detection after immunomagnetic capture	[6,40,41,42]

^a^ IVD approved by the FDA in 2014 [29]. ^b^ IVD approved by the FDA in 2016 [30]. ^c^ It is recommended to estimate TAG-72 along with other markers, especially CEA [32]. ^d^ FDA approved in 2002 for pancreatic cancer monitoring [35,43]. Now, it is also used for CRC monitoring [32]. It is recommended to estimate CA 19-9 along with other markers, especially CEA [33]. ^e^ FDA approved in 1995 [35]. ^f^ Companion device approved by the FDA in 2012 [36]. It was initially approved in 2012 and indicated for CRC patients treated with cetuximab [44]. ^g^ Companion device approved by the FDA in 2015 [37] ^h^ and 2017 [38]. ^i^ Companion device approved by the FDA in 2017 [39]. ^j^ FDA approved in 2005 for the outcome of metastatic breast cancer patients [45]. Now, it is also used for metastatic CRC [6,40,41,42] and metastatic prostate cancer [46] monitoring. Abbreviation**:** (ANXA3): annexin A3; (CA): carbohydrate antigen; (CEA): carcinoembryonic antigen; (CLEC4D): C-type lectin domain family 4 member D; (CRC): colorectal cancer; (DNA): deoxyribonucleic acid; (ELISA): enzyme-linked immunosorbent assay; (FDA): Food and Drug Administration; (FFPE): formalin-fixed paraffin-embedded; (FIT): fecal immunochemical test; (IL2RB): interleukin-2 receptor subunit beta; (IVD): in vitro diagnostic; (KRAS): Kirsten rat sarcoma viral oncogene homolog; (NA): not apply; (LMNB1): lamin-B1; (NRAS): neuroblastoma rat sarcoma viral oncogene homolog; (PCR): polymerase chain reaction; (PRRG4): proline-rich and Gla Domain 4; (qPCR): quantitative polymerase chain reaction or real-time polymerase chain reaction; (RNA): ribonucleic acid; (TAG-72): tumor-associated glycoprotein; (TNFAIP6): tumor necrosis factor-inducible gene 6 protein; (TPS): tissue polypeptide specific antigen; (VNN1): pantetheinase.

**Table 2:** Compiled list of proposed CRC blood biomarkers.

Type of Biomarker		Biomarker	Clinical Purpose			Ref.
			Diagnostic	Prognosis	Therapeutic Response Evaluation	
Genetic	Genes	SEPT9	X			[5]
	Mutation gene	APC, KRAS, TP53	X	X	X	[6,7]
Transcript	mRNA	CEA, cytokeratin 20, survivin, EGFR	X	X		[8,9,10]
Epigenetic	Hypermethylated DNA	MLH1, DAPK, RUNX3, ALX4, SEPT9, Vimentin, NEUROG1	X			[4,11]
		HLTF, HPP1, DFNA5		X		[6]
	Hypomethylated DNA	LINE-1	X			[5]
	miRNA	miRNA-15b, miRNA-18a, miRNA-19b, miRNA-20a, miRNA-21, miRNA-29a, miRNA-29b, miRNA-155, miRNA-194, miRNA-221, miRNA-335, miRNA-365, miRNA-1290	X			[4,5,6]
		miRNA-141, miRNA-200c		X		[4]
		miRNA-27b, miRNA-130b, miRNA-148a, miRNA-326, miRNA-484			X	[6]
		miRNA-19a, miRNA-106a	X		X	[5,6]
	Histones modification	H3K9me3, H4K20me3, H3K27me3	X			[5]
	Other non-coding RNA	NEAT1_v1, NEAT1_v2	X			[5]
Protein	Antibodies	Autoantibody-p53, Anti-p53, Anti-IMPDH2, Anti-MDM2, Anti-MAGEA4, FnIgA, FnIgG, Autoantibody-GTF2B, Autoantibody-MAPKAPK3, Autoantibody-PIM1, Autoantibody-PKN1, Autoantibody-SRC, Autoantibody-STK4, Autoantibody-SULF1	X			[5,47,48]
	Proteins	RBP4, THBS, TFF3, CEA, COL3A1, COL10A1, EGFR, CA11-19, MIC-1, GDF15, IL-6, IL-8, AZGP1, Angiopoetin-2, CL-L1, M-ficolin, MAp44, IGFBP2, DKK3, PKM2, CA19-9, CA50, CA72-4, p53, sFasL, VEGF	X			[5,6]
Cell	Circulating tumor cell (CTC)	CTC		X		[6]

Abbreviation: (Anti-IMPDH2 ): anti-inosine monophosphate dehydrogenase 2; (Anti-MAGEA4): melanoma-associated antigen 4; (Anti-MDM2): anti-mouse double minute 2 homolog; (ALX4): homeobox protein aristaless-like 4 gene; (APC): adenomatous polyposis coli; (AZGP1): zinc-alpha-2-glycoprotein; (CA): carbohydrate antigen; (CEA): carcinoembryonic antigen; (CL-L1): collectin liver 1; (COL3A1): collagen alpha-1(III) chain precursor; (COL3A1): collagen alpha-10(III) chain precursor; (DAPK1): death-associated protein kinase 1 gene; (DFNA5): deafness-associated tumor suppressor; (DKK3): dickkopf-related protein 3; (EGFR): epidermal growth factor receptor; (FnIgA): fusobacterium nucleatum immunoglobulin A; (FnIgG): fusobacterium nucleatum immunoglobulin G; (GDF15): growth/differentiation factor 15; (GTF2B): general transcription factor IIB; (H3K9me3): trimethylations of lysine 9 on histone 3; (H3K27me3): trimethylations of lysine 27 on histone 3; (H4K20me3): trimethylations of lysine 20 on histone 4; (HLTF): helicase like transcription factor; (HPP1): familial progressive hyperpigmentation 1; (IGFBP2): insulin-like growth factor-binding protein 2; (IL-6): interleukin-6; (IL-8): interleukin-8; (KRAS): Kirsten rat sarcoma viral oncogene homolog; (LINE-1): long interspersed nuclear elements 1; (MAp44): mannose-binding lectin-associated protein; (MAPKAPK3): MAP kinase-activated protein kinase 3; (MIC-1): macrophage inhibitory cytokine 1; (MLH1): mutL homolog 1; (NEAT1_v1): nuclear-enriched abundant transcript 1 v1; (NEAT1_v2): nuclear-enriched abundant transcript 1 v2; (NEUROG1): neurogenin-1 gene; (PIM1): Pim-1 proto-oncogene, serine/threonine kinase; (PKN1): protein kinase N1; (PKM2): pyruvate kinase isozymes M2; (RBP4): retinol binding protein 4; (RUNX3): runt-related transcription factor 3 gene; (sFasL): soluble Fas Ligand; (SRC): SRC proto-oncogene, non-receptor tyrosine kinase; (STK4): serine/threonine kinase 4; (SULF1): sulfatase 1; (THBS): thrombospondin 1; (TFF3): trefoil factor 3; (TP53): tumor protein p53; (VEGF): vascular endothelial growth factor.

**Table 3:** Electrochemical biosensors for the determination of CRC-associated nucleic acids.

Biomarker	Electrode Support	Detection Method/ Redox Probe	Dynamic Linear Range	Limit of Detection (LOD)	Test Matrix	Ref.
Gene sequence-associated with CRC	GCE/(CeO_2_-CHIT)	DPV/Methylene blue	1.59 × 10^−11^ to 1.16 × 10^−7^ M	1.0 × 10^−11^ mol L^−1^	NA	[95]
KRAS	Au	Amperometry/ HQ	1.17 × 10^−11^ to 1.17 × 10^−7^ M	5.85 × 10^−12^ M	NA	[98]
	GCE	DVP/AuCl_4_^-^	0.1 to 100 pM	30 fM	Cell line SW480	[99]
	Phthalocyanine-BODIPY dye/Graphite	DVP/No reported	1.54 × 10^−4^ to 1.92 × 10^−2^ µg mL^−1^	2.06 × 10^−6^ µg mL^−1^	Blood	[100]
	A1/PtTiO_2_-reduced graphene oxide		3.07 × 10^−7^ to 3.84 × 10^−3^ µg mL^−1^	8.67 × 10^−10^ µg mL^−1^		
	A2/PtTiO_2_-reduced graphene oxide		3.84 × 10^−8^ to 0.48 µg mL^−1^	2.94 × 10^−5^ µg mL^−1^		
KRAS mutation (KRAS G12D)	Au	DPV/Methylene blue	0.01 fM to 1 pM	2.4 aM	Plasma	[106]
	Au	SWV/Methylene blue	5.92 pM to 10 nM for t-DNA 100 pM to 10 nM for M-DNA	Not reported	Serum	[107]
BRAF mutation (BRAF V600E)	SPCE	DPV/AA	50–0.8% of V600E alleles	Not reported	Cell line HT29	[110]
CEACAM5	Au	Not reported	Not reported	Not reported	NA	[114]
	PET/VA-MWCNTs-COOH	CV/Methylene blue	50 to 250 µM	0.92 µM	Cell line T84	[113]
5-hmC MGMT	SPCE	Amperometry/HQ	77 to 7500 pM (ProtA-polyHRP_80_)	23 pM	Cell lines SW480 SW620 Colorectal tissues	[115]
			44 to 5000 pM (Histostar)	13 pM		
miRNA-21	Au/AuNPs	Amperometry/ hemin-G-quadruplex complex	5 to 5000 pM	3.96 pM	Cell line HT29	[118]
	SPAuE	DPV/[Fe(CN)_6_]^4−/3−^	1.0 pM to 10 nM	1.0 pM	Cell line SW-48 Serum	[119]

Abbreviations: (A1): 2,6-bis((E)-2-(furan-2-yl)vinyl)-4-(4,6,8-trimethylazulen-1-yl)pyridine; (A2): 2,6-bis((E)-2-(thiophen-3-yl)vinyl)-4-(4,6,8-trimethylazulen-1-yl)pyridine; (AA): ascorbic acid; (Au): gold electrode; (AuCl^4-^): tetrachloroaurate ion; (BRAF): v-Raf murine sarcoma viral oncogene homolog B; (CEACAM5): carcinoembryonic antigen-related cell adhesion molecule 5; (CeO_2_-CHIT): cerium oxide-chitosan composite; (CV): cyclic voltammetry; (DPV): differential pulse voltammetry; (GCE): glassy carbon electrode; ([Fe(CN)_6_]^4−/3−^): redox couple ferrocyanide/ferricyanide; (HQ): hidroquinone; (KRAS): kirsten rat sarcoma viral oncogene homolog; (5-hmC): 5-hydroxymethylcytosine; (M-DNA): mutant DNA; (MGMT): O^6^-methylguanine-DNA methyltransferase; (miRNA-21): microRNA-21; (VA-MWCNTs-COOH): vertically aligned carboxylated multi-walled carbon nanotubes; (NA): not apply; (PET): polyethylene terephthalate; (SPAuE): screen-printed gold electrode; (SPCE): screen-printed carbon electrode; (SWV): square wave voltammetry; (t-DNA): total DNA.

**Table 4:** Electrochemical biosensors for the determination of CRC-associated proteins.

Biomarker	Electrode Support	Detection Method/ Redox Probe	Dynamic Linear Range	Limit of Detection (LOD)	Tested Matrix	Ref.
CEA	Au	DPV/HQ	0 to 200 ng mL^−-1^	0.2 ng mL^−1^	Serum	[123]
	(FTO) glass	EIS/(I^−^/I^3−^)	0.125 to 7.5 pg mL^−1^	0.0832 pg mL^−1^	Urine	[124]
	(FTO) glass	Voltammetry/(I^−^/I^3−^)	10 ng mL^−1^ to 100 µg mL^−1^	0.14 ng mL^−1^	Urine	[125]
	(FTO) glass	Power/(I^−^/I^3−^)	0.025 to 0.75 ng mL^−1^	0.10 pg mL^−1^	Urine	[126]
Autoantibodies-p53	SPCE	Amperometry/HQ	1.1 to 5 U mL^−1^	0.34 U mL^−1^	Serum	[131]
	SPAuE/(Au@NPFe_2_O_3_ NC)	Amperometry/TMB	0.02 to 14 U mL^−^^1^	0.02 U mL^−1^	Serum	[132]
p53	ITO/Star_PGMA_	EIS/[Fe(CN)_6_]^4−/3−^	0.02 to 4 pg mL^−1^	7 fg mL^−1^	Serum	[134]
FAM134B	SPCE/Extravidin	DPV/[Fe(CN)_6_]^4−/3−^	0.01 to 100 ng μL^−1^	10 pg μL^−1^	Cell lines SW480 SW48 HCT116 Serum	[138]
EpCAM	Au/AgNPs-CHIT	Amperometry/(4-TBC)	Not reported	2.7 pg mL^−1^	Peripheral blood	[141]
	Au/AgNPs-PVA	Amperometry/(4-TBC)	2 to 2000 pg mL^−1^	0.8 pg mL^−1^	Peripheral blood	[142]
LRG1	Au	EIS/[Fe(CN)_6_]^4−/3−^	0 to 0.25 μg mL^−1^	0.025 μg mL^−1^	Plasma	[145]
CXCL5	GCE	Amperometry/Hydrazine	0.1 to 10 ng mL^−1^	0.078 ng mL^−1^	Cell line HT29 Serum	[147]
CA19-9	SPIDE/CNO-GO	EIS/Phosphate Buffer	0.3 to 100 U mL^−1^	0.12 U mL^−1^	Cell line HT29	[151]
ET-1	Au	EIS/[Fe(CN)_6_]^3^^−^^/4^^−^	2 to 100 pg mL^−^^1^	0.34 pg mL^−^^1^	NA	[162]
IL-13Rα2	SPCE	Amperometry/HQ	3.9 to 100 ng mL^−^^1^	1.2 ng mL^−^^1^	Cell lines SW480 SW620 KM12C KM12SM	[152]
	SPCE/pABA/strep	Amperometry/HQ	2.7 to 100 ng mL^−^^1^	0.8 ng mL^−^^1^	Cell lines SW480 SW620 KM12C KM12SM Colorectal tissues	[153]
RANKL	SPCE/pABA/strep	Amperometry/HQ	10.4 to 1000 ng mL^−^^1^	3.1 ng mL^−^^1^	Serum	[154]
E-cad	SPCE	Amperometry/HQ	0.5 to 25 ng mL^−^^1^	0.16 ng mL^−^^1^	Cell lines SW480 SW620 KM12C KM12SM Colorectal tissues	[160]
CDH17	SPCE	Amperometry/HQ	4.8 to 1000 ng mL^−^^1^	1.43 ng mL^−^^1^	Cell lines SW480 SW620 KM12C KM12SM Colorectal tissues	[161]
IL-6	GCE/pABA/pATP/AuNPs	EIS//[Fe(CN)_6_]^4−/3−^	5 pg mL^−1^ to 100 ng mL^−1^	1.6 pg mL^−1^	Serum	[164]

Abbreviations: (4-TBC): 4-tert-butylcatechol; (Au): gold electrode; (Au@NPFe_2_O_3_ NC): gold-loaded nanoporous iron oxide nanocube; (AgNPs-CHIT): silver NP (AgNPs) covered by chitosan; (CA): carbohydrate antigen; (CEA): carcinoembryonic antigen; (CDH17): cadherin-17; (CHIT); (AgNPs-PVA): Silver NP covered with polyvinyl alcohol (PVA); (CXCL5): chemokine (C–X–C motif) ligand 5; (DPV): differential pulse voltammetry; (E-cad): E-cadherin; (EIS): electrochemical impedance spectroscopy; (EpCAM): epithelial cell adhesion molecule; (ET-1): peptide endothelin 1; (FAM134B): family with sequence similarity 134 member B; ([Fe(CN)_6_]^3−/4−^): redox couple ferrocyanide/ferricyanide; (FTO): fluorine-doped tin oxide glass; (GCE): glassy carbon electrode; (GCE/pABA/pATP/AuNPs): glassy carbon electrode modified with p-aminobenzoic acid (pABA), p-aminothiophenol (pATP), and AuNP; (HQ): hydroquinone; (I^−^/I^3−^): redox couple iodide/triiiodide; (IL-6) interleukin-6; (IL-13Rα2): interleukin-13 receptor Rα2; (RANKL): ligand receptor activator nuclear factor-κB; (LRG1): leucine-rich α-2-glycoprotein 1; (SPAuE): screen-printed gold electrodes; (SPCE): screen-printed carbon electrodes; (SPCE/pABA/strep): immobilized streptavidin on p-aminobenzoic acid (pABA) grafted at an SPCE; (SPDE/GO) and (SPIDE/CNO-GO): screen-printed interdigitated electrode (SPIDE) modified with carbon nano-onions (CNOs) and graphene oxide (GO); (StarPGMA-ITO): star-shaped poly(glycidylmethacrylate)-modified indium tin oxide (ITO) electrode; (TMB): tetramethylbenzidine.

**Table 5:** Electrochemical biosensors for the determination of CRC-associated cells.

Biomarker	Electrode Support	Detection Method/ Redox Probe	Dynamic Linear Range [cells mL^−1^]	Limit of Detection (LOD) [cells mL^−1^]	Test Matrix	Ref.
Caco2	SPCE	Amperometry/HCl	1 × 10^4^ to 5 × 10^4^ cells	2.2 × 10^2^ cells	Cell culture	[172]
HCT116	SPAuE	CV/[Fe(CN)_6_]^3−^	1 to 100	7	Cell culture	[173]
	GCE/(NH_2_/GO)	EIS/[Fe(CN)_6_]^3−/4−^	5 × 10^2^ to 5 × 10^6^	100	Cell culture	[174]
DLD-1	GCE/CNSs	EIS/[Fe(CN)_6_]^3−/4−^	1.25 × 10^2^ to 1.25 × 10^6^	40	Cell culture	[177]
	GCE/BSA-AgNFs	EIS/[Fe(CN)_6_]^3−/4−^	1.35 × 10^2^ to 1.35 × 10^7^	40	Cell culture	[180]
CT26	SPCE/AuNPs/SBA-15-pr-NH_2_	EIS/[Fe(CN)_6_]^3−/4−^	10 to 1 × 10^5^	2	Cell culture	[181]
HT29	GCE/KCC-1- NH_2_	SWV/DPV/[Fe(CN)_6_]^3−/4−^	50 to 1.2 × 10^4^	Not reported	Cell culture	[182]
	Au/MWCNTs-COOH	CC-PSA/Trp	0.0001 to 10 mM	64 pM	Cell culture	[183]
Colo320 HCT116	Carbon/Gold	Amperometry/ p-aminophenol	0.25 to 10 µg mL^−1^	Not reported	Cell culture	[184]

Abbreviations: (Au): gold electrode; (AuNPs/SBA-15-pr-NH_2_): mesoporous silica SBA-15 with 3-aminopropyltriethoxysilane and gold nanoparticles; (BSA-AgNFs): BSA-incorporated Ag nanoflowers; (CC-PSA): constant current-potentiometric stripping analysis; (CNSs): carbon nanospheres; (CV): cyclic voltammetry; (DPV): differential pulse voltammetry; (EIS): electrochemical impedance spectroscopy; ([Fe(CN)_6_]^3−/4−^): redox couple ferrocyanide/ferricyanide; (GCE): glassy carbon electrode; (HCl): chloride acid; (KCC-1-NH_2_): amine-functionalized fibrous nano-silica; (MWCNTs-COOH): carboxylated multi-wall carbon nanotubes; (NH_2_/GO): amine-functionalized graphene oxide; (SPAuE): screen-printed gold electrode; (SPCE): screen-printed carbon electrode; (Trp): tryptophan; (SWV): square wave voltammetry.
